# Origin of the Magnetization Anisotropy of Superparamagnetic Beads

**DOI:** 10.1002/smll.202508156

**Published:** 2026-01-08

**Authors:** Sebastian Belau, Fabian Welzel, Dominik J. Kauert, Aidin Lak, Ralf Seidel

**Affiliations:** ^1^ Peter Debye Institute For Soft Matter Physics University of Leipzig Leipzig Germany; ^2^ Institute For Electrical Measurement Science and Fundamental Electrical Engineering and Laboratory for Emerging Nanometrology (LENA) TU Braunschweig Braunschweig Germany

**Keywords:** magnetic anisotropy, magnetic tweezers, magnetorelaxometry, nanoparticles, single molecule, superparamagnetic beads

## Abstract

Superparamagnetic beads are used in single‐molecule magnetic tweezers experiments to investigate the mechanics and dynamics of biomolecules. The beads exhibit a weak anisotropy, such that they align with the applied magnetic field. This allows to rotate the beads and thus to twist of attached biomolecules. To quantitatively understand the origin of the magnetization anisotropy, we use high‐speed magnetic tweezers experiments, numerical simulations, as well as fluxgate magnetorelaxometry measurements. We find that Brownian orientation fluctuations of the beads occur up to cut‐off frequencies of ∼100 Hz being only weakly dependent on the applied field, which superimpose the dynamics of attached biomolecules. When simulating the equilibrium magnetization of single beads as ensembles of ∼10^5^ anisotropic and randomly oriented superparamagnetic nanoparticles, a single anisotropy axis is formed, which matches the magnitude of the experimental results. The time scale of the magnetization relaxation spans several orders of magnitude. Overall, our data reveal that the magnetic beads contain randomly oriented magnetic domains with a rather wide size distribution in which the bead anisotropy is the finite residual of the sum of the nanoparticle anisotropies. It is thus an inherent property of magnetic beads and needs to be considered in high‐resolution measurements of magnetic tweezers.

## Graphical abstract

Superparamagnetic beads are widely employed to study the mechanics of biomolecules with magnetic tweezers. It is shown that the residual anisotropy of the beads is due to averaging over a finite number of randomly oriented nanoparticles contained in the beads. Pinning of the anisotropic beads in the field causes low and high‐frequency noise in magnetic tweezers measurements, and ways to overcome this resolution limit are discussed.

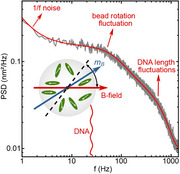

## 1 Introduction

**FIGURE 1:**
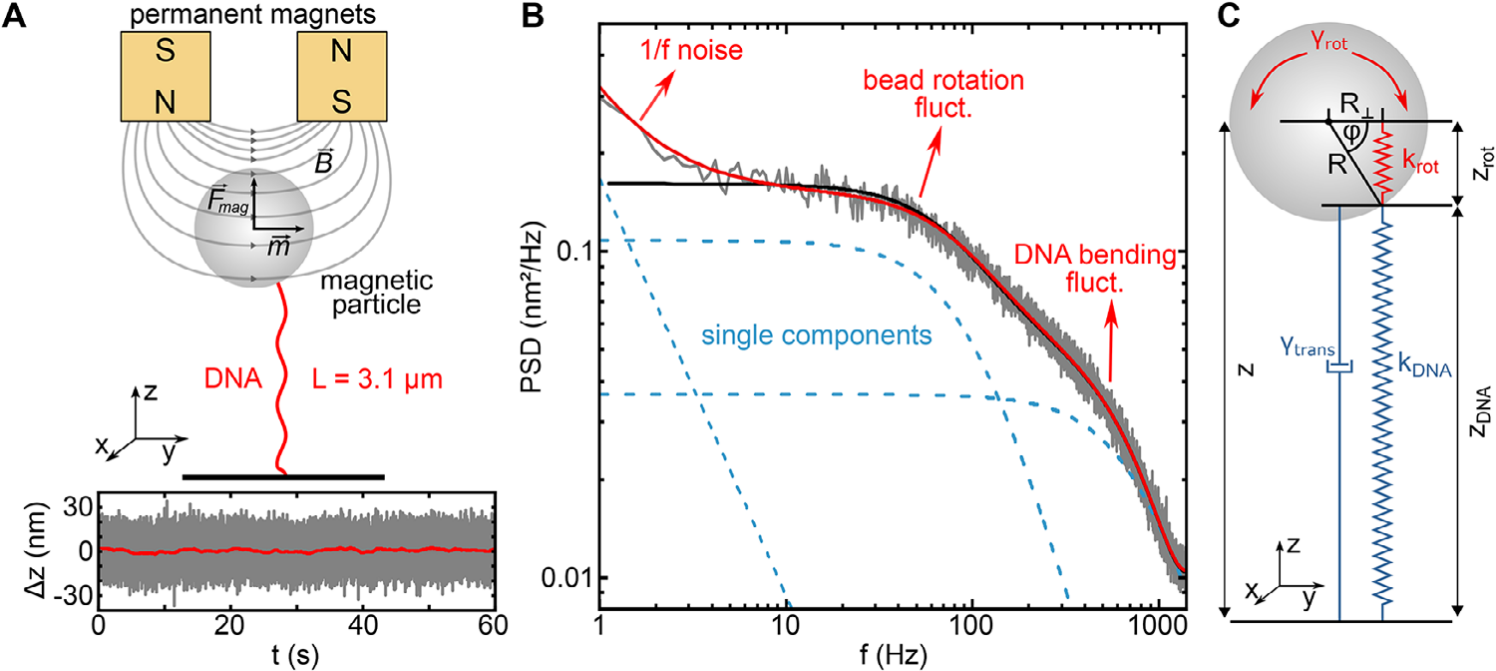
(A) Scheme of the DNA length measurements using magnetic tweezers (top). Time trajectory of position changes along the z‐coordinate (relative to an equilibrium position) of a 1‐µm magnetic bead stretching a 3.1‐µm long DNA molecule at a force of 7 pN (bottom). Positions were recorded at 2841 Hz (grey) and smoothed with a sliding average filter to 1 Hz (red). (B) PSD of the time trajectory shown in A (grey). The data is a fit employing a double Lorentzian according to two coupled fluctuation modes with (red) and without (black) an additional 1/*f* noise component. The light blue dashed lines correspond to the individual components of the double Lorentzian fit and the 1/*f* noise. (C) Coupled oscillator model for the observed PSDs. Axial position fluctuations of the bead along z arise from the coupling of DNA length fluctuations and rotational fluctuations of the magnetic bead [13].

Magnetic tweezers have become widely used mechanical tools for stretching [1, 2, 3, 4] and twisting [5, 6, 7, 8, 9] single biomolecules as well as for manipulating cellular machines [10, 11]. In a widespread magnetic tweezers design, a single biomolecule is attached at one end to a glass surface and at the other end to a magnetic bead (Figure 1A). A pair of permanent magnets produces a strong magnetic field gradient and allows to pull the beads along the gradient direction, stretching the attached biomolecule. Due to the intrinsic magnetic anisotropy of the beads, they align with the magnetic field, allowing the twisting of the biomolecule by rotating the magnets. By tracking the bead position in three dimensions with a camera, the end‐to‐end distance of the biomolecule can be measured and monitored, revealing conformational changes like the untwisting of DNA [6] or the unwinding of DNA hairpins by molecular motors [3].

The pulling forces induced by the external field on the magnetic beads are well understood and can be quantified using the off‐axis/horizontal thermal fluctuations of the beads in the pulling configuration [12, 13, 14]. Furthermore, they can be obtained from measurements of the magnetic moment of the beads [13] and finite‐element simulations of the magnetic field for a particular magnet configuration [15, 16]. The torque that is generated during biomolecule twisting can also be measured using different approaches [5, 15, 17]. Torque generation is, however, not understood on the theoretical level since the anisotropy of the magnetization of a bead is by 2–3 orders of magnitude smaller than the total magnetic moment [11, 13]. The commercial magnetic beads that are employed in magnetic tweezers experiments are typically comprised of maghemite (𝛾‐Fe_2_O_3_) NPs with a radius of about 4 nm [18] embedded in a polymer matrix. The individual NPs have their own anisotropy. The magnetocrystalline anisotropy constant of maghemite is about 5 kJ/m^3^ [19, 20, 21, 22]. For NPs the anisotropy is additionally significantly increased by the shape and surface properties [23]. Due to a random orientation of the NPs and their large number inside a single bead (around 3×10^5^ particles per 1 µm‐sized MyOne bead and 2×10^6^ particles per 2.8 µm‐sized M280 bead, see Methods), one might expect that their individual anisotropies cancel each other, leading to a vanishing effective anisotropy of a bead as a whole. Nonetheless, in experiments, a pronounced anisotropy of the beads is always observed, whose origin is not resolved yet.

As pointed out above, the magnetic beads align with their anisotropy axis along the field lines (Figure 1A). While the beads are able to freely rotate around the field direction, rotation around perpendicular axes is strongly constrained. Randomly attached DNA molecules thus frequently display an off‐center anchoring in magnetic tweezers experiments, where the DNA axis is displaced along the field direction from the center of the magnetic bead (Figure 1A). It has been shown [13], that constrained rotational bead fluctuations about the alignment axis, in combination with the off‐center attachment, cause fluctuations of the bead position along the stretching direction (z‐direction, see Figure 1B). These fluctuations couple to the DNA length fluctuations along the z‐direction, leading to an overall noise increase in DNA length measurements [13]. This inherently affects the spatio‐temporal resolution of magnetic tweezers, where conformational changes of interest often occur at a base‐pair length scale and sub‐millisecond time scale.

The dynamics of the rotational fluctuations have so far not been characterized [11, 13]. To explore their characteristic time scales, we conducted in this study DNA length measurements at kHz framerates combined with power spectral density (PSD) analysis. The contributions of the rotational fluctuations occurred on top of the DNA length fluctuations throughout the range of applied forces up to characteristic cut‐off frequencies of 100–200 Hz for 1‐µm and 2.8‐µm beads. Furthermore, we observed 1/f‐like noise at low frequencies.

To shed light on the anisotropic nature of the magnetic beads, we simulated the equilibrium magnetization of entire beads in the magnetic field. We modelled them as an ensemble of isolated randomly oriented magnetic NPs, which always yielded a single pronounced anisotropy axis. The torsional stiffness of the simulated beads in the field reproduced the experimentally measured values. Using fluxgate magnetorelaxometry (MRX) [24], we furthermore observed that the magnetization of the beads relaxes on different time scales ranging from nanoseconds to seconds. Altogether, this supports that the magnetic beads consist of randomly oriented magnetic NPs with wide size distributions. The bead magnetization anisotropy stems from the residual average magnetization anisotropy of the randomly oriented NPs. It is thus inherent to the nanoscale structure of the magnetic bead particles, and we discuss its consequences for magnetic tweezers measurements.

## 2 Results

### 2.1 Ultrafast Measurements of Axial Position Fluctuations of Magnetic Beads

Our measurements of the axial position fluctuations of magnetic beads used single DNA molecules of 9546 bp length (contour length of ∼3.1 µm) that were tethered at one end to the surface of a fluidic cell and at the other end to a superparamagnetic bead. To generate force parallel to the optical axis (z‐direction, see Figure 1A), we used a pair of magnets with vertical magnetizations, being aligned in an anti‐parallel orientation. Using a small gap of 0.3 mm between them [16], allowed to achieve maximum stretching forces of ∼13 pN for 1‐µm beads (MyOne) and ∼55 pN for 2.8‐µm beads (M280), respectively, with actual values differing between individual beads.

To identify different modes of the position fluctuations of DNA‐tethered magnetic beads, a high spatio‐temporal resolution as well as a large bandwidth were required. To achieve this, we employed high‐speed particle tracking at 2800 kHz with nanometer resolution [25]. We next measured long position trajectories (900 s) of DNA‐tethered magnetic beads at constant force (Figure 1A). To characterize axial position fluctuations of the magnetic beads, we calculated the single‐sided power spectral densities (Figure 1B). If governed by strongly overdamped DNA length fluctuations, one would expect a simple Lorentzian‐shaped spectrum [26]:

(1) $$PSD_{Z,DNA}\left(f\right)=\frac{4k_{B}T\gamma_{trans}}{k_{DNA}^{2}}\frac{1}{1+{\left(f/f_{DNA}\right)}^{2}}$$

with $k_{B}$ being the Boltzmann constant, T the temperature, $k_{\mathrm{DNA}}$ the axial entropic stiffness of the DNA, $\gamma_{\mathrm{trans}}$ the translational drag coefficient of the magnetic bead and $f_{\mathrm{DNA}}=k_{\mathrm{DNA}}/2\pi \gamma_{\mathrm{trans}}$   the characteristic cut‐off frequency (see Methods). The mean‐square displacement over the entire frequency range is given by $\left\langle z_{DNA}^{2}\right\rangle =k_{B}\;T/k_{DNA}$. On a double logarithmic scale, the expected Lorentzian exhibits a plateau below and a $1/f^{2}$ decay above $f_{\mathrm{DNA}}$, respectively (blue dashed lines in Figure 1B). When inspecting the PSDs from the experimental data, however, the spectra exhibited a more complex behavior. They typically contained two transition points, indicating the presence of two different fluctuation modes as well as a low‐frequency noise component with an approximate 1/f decay (Figure 1B).

To analyze the experimentally obtained PSDs, we described our DNA tethered beads as a system of two coupled overdamped oscillators, one comprising the DNA length fluctuations (with $k_{\mathrm{DNA}}$, $\gamma_{\mathrm{trans}}$) and the other the rotational bead fluctuations with the field‐dependent torsional spring constant $k_{\mathrm{tor}}$ and the rotational drag coefficient $\gamma_{\mathrm{tor}}$ (Figure 1C). For an off‐center attachment R_⊥_ of the DNA axis with respect to the bead center, the rotational fluctuations change the height of the DNA attachment point and thus couple with the DNA length fluctuations. One can show that the PSD of such a coupled system is given by a simple sum of two Lorentzians [13] (see Methods, Equations S3 and S4) for which the two characteristic frequencies $f_{\pm }^{coupl}$ can be approximated by (see Note S1, Equation S25):

(2) $$f_{\pm }^{coupl}\approx \left\{\begin{array}{c} f_{DNA}\left(1+\frac{6R_{⊥}^{2}}{8R^{2}}\right)\\ f_{rot}\left(1-\frac{6R_{⊥}^{2}}{8R^{2}}\right) \end{array}\right.$$

where 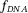 and 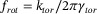 are the cut‐off frequencies of the individual uncoupled oscillators and *R* is the bead radius. Thus, $f_{+}^{coupl}$ is dominated by the DNA length fluctuations and increases from 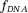 with increasing R⊥, while $f_{-}^{coupl}$ is dominated by the rotational fluctuations and slowly decreases from 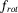 with increasing R⊥ (see Figure S1). Notably, the mean‐square displacement of the coupled oscillator equals the sum of the mean‐square amplitudes of the individual oscillators, independent of coupling.

To account for low‐frequency drift, we added an additional 1/f term (see Methods, Equation S6) to the double Lorentzian model function (Equations S3 and S4). Fitting of the experimentally obtained PSDs with the three‐component equation provided an excellent agreement over the entire frequency range (Figure 1B and Note S2, Figure S2). As best‐fit parameters, we obtained $k_{\mathrm{DNA}}$, $\gamma_{\mathrm{trans}}$, $k_{\mathrm{tor}}$, $\gamma_{\mathrm{tor}}$, A and α. From these the root‐mean‐squared displacement (RMS) of the bead fluctuations $\sqrt{\left\langle z^{2}\right\rangle }$ as well as the cut‐off frequencies of the coupled system $f_{\pm }^{coupl}$ were calculated.

### 2.2 Dynamics of the Rotational Fluctuations of Different Magnetic Beads

**FIGURE 2:**
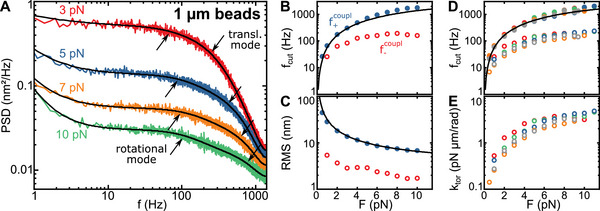
(A) Experimentally obtained PSDs of the axial fluctuations of a 1‐µm DNA‐tethered bead at different stretching forces (coloured lines). Double Lorentzian fits to the data incl. 1/f noise are shown in black. Downward arrows indicate the translational fluctuation mode and upward arrows the rotational fluctuation mode. (B and C) Cut‐off frequencies $f_{+}^{coupl}$ and $f_{-}^{coupl}$ as well as the RMS amplitudes $\sqrt{\left\langle z_{DNA}^{2}\right\rangle }$ and $\sqrt{\left\langle z_{rot}^{2}\right\rangle }$ of the two fluctuation modes for the data set shown in A. Blue‐filled circles ($f_{+}^{coupl})$ correspond to the mode that is dominated by DNA length fluctuations and red open circles $f_{-}^{coupl}$ to the mode that is dominated by rotation fluctuations of the bead. Black lines show the theoretical expectations for pure DNA length fluctuations. (D, E) Cut‐off frequencies and torsional stiffness as a function of the applied force obtained for several DNA‐tethered beads (subset of *N*  =  5, as indicated by the different colours). Closed and open circles correspond to the two fluctuation modes being dominated by DNA length and bead rotation fluctuations, respectively.

**FIGURE 3:**
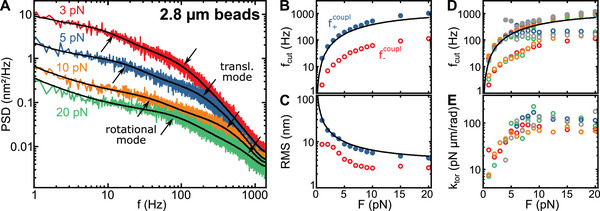
(A) Experimentally obtained PSDs of the axial fluctuations of a 2.8‐µm DNA‐tethered magnetic bead at different stretching forces (coloured lines). Double Lorentzian fits to the data incl. 1/f noise is shown in black. Downward arrows indicate the translational fluctuation mode and upward arrows the rotational fluctuation mode. (B and C) Cut‐off frequencies $f_{+}^{coupl}$ and $f_{-}^{coupl}$ as well as the RMS amplitudes $\sqrt{\left\langle z_{DNA}^{2}\right\rangle }$ and $\sqrt{\left\langle z_{rot}^{2}\right\rangle }$ of the two fluctuation modes for the data set shown in (A). Blue‐filled circles ($f_{+}^{coupl})$ correspond to the mode that is dominated by DNA length fluctuations and red open circles ($f_{-}^{coupl})$ to the mode that is dominated by rotation fluctuations of the bead. Black lines show the theoretical expectation for pure DNA length fluctuations. (D and E) Cut‐off frequencies and torsional stiffness as a function of the applied force obtained for several DNA‐tethered beads (*N *= 5, as indicated by the different colours). Closed and open circles correspond to the two fluctuation modes being dominated by DNA length and bead rotation fluctuations, respectively.

Using the methodology described above, we characterized the contribution of rotational fluctuations to DNA length measurements employing 1‐µm and 2.8‐µm magnetic beads. To this end, we recorded trajectories of the axial positions of DNA‐tethered beads at different forces and calculated the corresponding PSDs. At higher forces, the two fluctuation modes could be clearly identified (Figures 2A and 3A) while at lower forces the cut‐off frequencies of the two modes approached each other such that discrimination became more difficult.

For 1‐µm beads, the dominating fluctuation mode with the largest RMS amplitude, exhibited the larger cut‐off‐frequency $f_{+}^{coupl}$. While increasing the applied force from 0.5 to 10 pN, the RMS amplitude decreased by about one order of magnitude, and the cut‐off frequency increased by about two orders of magnitude (Figure 2B,C). To support that this mode is dominated by DNA length fluctuations (see Methods), we obtained the force‐dependent spring constant $k_{\mathrm{DNA}}$ from the extensible WLC model [27] and calculated theoretical values for $\left\langle z_{DNA}^{2}\right\rangle$ and $f_{\mathrm{DNA}}$, which showed good agreement with the experimental results for $f_{+}^{coupl}$ (Figure 2B,C, black lines). The mode with the lower cut‐off frequency could thus be attributed to the rotational fluctuations of the bead. The corresponding RMS amplitude decreased from 5.4 nm at an applied force of 1 pN to 1.7 nm at 10 pN, while its cut‐off frequency $f_{-}^{coupl}$ increased only slightly to a plateau value of 100–200 Hz. Repeating the same experiment and analysis for several beads (N = 10), provided similar results (Figure 2D).

As additional proof of the model assumptions, we calculated the hydrodynamic radii R of the beads from the obtained values for $\gamma_{\mathrm{trans}}$ (Note S3 and Figures S3–S5). The average value of 0.51 ± 0.13 µm is in good agreement with the nominal bead size of 1‐µm (Figure S5A). This further supported that the high‐frequency mode can be faithfully assigned to linear DNA length fluctuations.

We furthermore determined the off‐center attachments R⊥ (see Methods and Figures S3 and S4) to obtain the torsional stiffnesses $k_{\mathrm{tor}}$ for the alignment of 1‐µm beads in the magnetic field (Figure 2E). $k_{\mathrm{tor}}$ was found to gradually increase at low forces/magnetic fields and started to saturate at high forces in agreement with previous reports [11, 13]. We also observed the expected large spread of $k_{\mathrm{tor}}$ for the individual beads. The mean torsional stiffness at 10 pN was 3.2 ± 0.7 pN µm/rad, which is of the same order of magnitude as previously reported values of 9.5 ± 2.2 pN µm/rad [11].

For larger 2.8‐µm beads, our observations were similar to those of the smaller beads. The high‐frequency fluctuation mode could again be attributed to DNA length fluctuations (Figure 3B,C). The RMS amplitude of the lower‐frequency mode from rotational fluctuations of the bead decreased from 8.7 to 2.7 nm when increasing the applied force from 10 to 20 pN. Its cut‐off frequency increased when increasing the applied force and plateaued around 100–200 Hz at higher forces (Figure 3B,D), covering a similar frequency range as the 1‐µm beads. The effective hydrodynamic radii *R* yielded a mean of 1.41 ± 0.31 µm, which was again in good agreement with the nominal bead size (Figures S5B and S6).

For the torsional stiffness $k_{\mathrm{tor}}=k_{\mathrm{rot}}R_{⊥}^{2}$ of 2.8‐µm beads in the magnetic field, we observed again a gradual increase at lower forces followed by a saturation around a torsional stiffness of 105 ± 45 pN µm/rad in agreement with previous studies [11, 13]. Also, for this bead type a high bead‐to‐bead variation was observed.

### 2.3 1/f‐Like Noise at Low Frequencies

**FIGURE 4:**
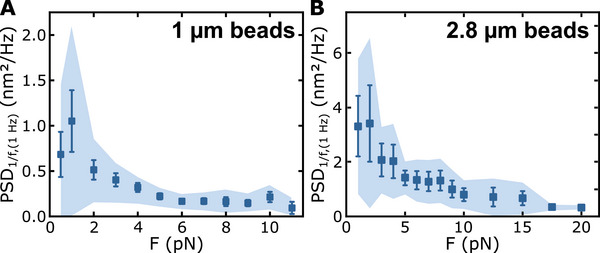
Mean magnitude of the 1/f‐like low frequency noise at 1 Hz for (A) 1‐µm beads (*N* = 10) and (B) 2.8‐µm beads (*N* = 5). The error bars represent the standard errors of the mean, and the shaded area the standard deviation. For the 2.8‐µm beads, a noise of the form $\mathrm{PS}D_{1/f}=A/f$  was considered, while for the 1‐µm beads a noise of the form $\mathrm{PS}D_{1/f}=A/f^{\alpha }$ was used, with A and α being free‐fitting parameters.

At frequencies below approximately 5 Hz, 1/f ‐like noise became visible in the PSD of the vertical/axial fluctuations and was included in the PSD fitting as an additional term $\mathrm{PS}D_{1/f}=A/f^{\alpha }$   (Equation S6) with A defining the amplitude of the low‐frequency noise. For the 2.8‐µm beads, the low‐frequency noise was well described by α  =  1 such that this parameter was fixed. For 1‐µm beads, we obtained α values in the range of 0.5 ≤ α ≤ 1.5. $\mathrm{PS}D_{1/f}$ at 1 Hz decreased for both bead types with increasing force and was more pronounced for 2.8‐µm beads compared to the 1‐µm beads (Figure 4). Both observations suggest that the observed 1/f‐like noise is not due to simple instrument drift that was insufficiently corrected by the reference particles.

When inspecting bead‐position trajectories recorded on short molecules with reduced DNA length fluctuations, we observed 1/f‐like noise not only along the axial direction but in a correlated manner also laterally along the magnetic field direction (Note S4 and Figure S7). It was however, not observed in the transverse direction to the field. It can thus be unambiguously attributed to small and slow orientation changes of the anisotropy axis that in turn lead to the observed low position fluctuation of the beads (see Discussion).

### 2.4 Simulating the Magnetization of Beads Provides a Well‐defined Anisotropy Axis

**FIGURE 5:**
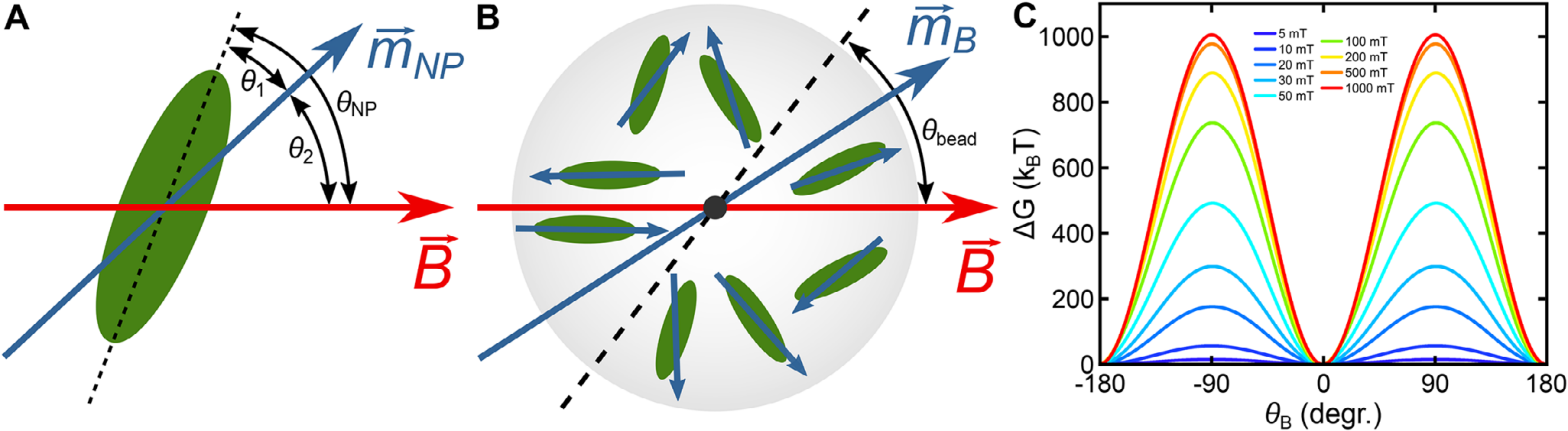
Simulations of the magnetization of a magnetic bead. (A) Scheme of the magnetic moment ${\vec{m}}_{NP}$ of a single magnetic nanoparticle (NP, green oval) whose anisotropy axis is located at an angle *θ*_*NP*_ with respect to the applied magnetic field $\vec{B}$. The applied field displaces the magnetic moment by an angle *θ*_1_ from the anisotropy axis. (B) Scheme of a superparamagnetic bead containing individual randomly oriented magnetic NPs (green ovals) together with their instantaneous magnetic moments in an applied magnetic field. For a given NP, the two orientations with minimum potential energy are shown in either the upper or the lower bead half. The magnetic moments of the NPs add up to yield the total magnetic moment of the bead ${\vec{m}}_{B}\left(B\right)$. (C) Simulated total free energy for the magnetization of a 1‐µm magnetic bead as a function of the bead orientation using *K* = 30 kJ/m^3^. For each curve, the minimum free energy was set to zero angle. The pronounced minima of the free energy function every 180° reveal the formation of a well‐defined anisotropy axis.

After characterizing the dynamics and the strength of the pinning of the beads in the magnetic field, we simulated the magnetization of single beads. To this end, we modelled the beads as an ensemble of randomly oriented anisotropic superparamagnetic NPs that do not mutually interact. The potential energy of a single superparamagnetic NP in an external magnetic field can be approximately described by the Stoner–Wohlfarth model [28]:

(3) $$U\left(B,\theta_{NP},\theta_{2}\right)=\frac{1}{2}\;KV\sin^{2}\left(\theta_{NP}-\theta_{2}\right)-MVB\cos\theta_{2}$$

where K is the anisotropy constant, M the volume magnetization of the material and V the particle volume. The magnetic moment of a superparamagnetic NP is defined by $m_{\mathrm{NP}}=\mathrm{MV}$, which has a constant magnitude but can adapt random orientations. *θ*_*NP*_ is the angle between the magnetic field $\vec{B}$ and the anisotropy axis of the NP, while *θ*_2_ is the angle between $\vec{B}$ and the magnetic moment ${\vec{m}}_{NP}$ of the NP (see Figure 5A). For a single free NP, the potential energy depends on the orientation of ${\vec{m}}_{NP}$ with respect to the anisotropy axis (left term) and the magnetic field $\vec{B}$ (right term). To reduce the computational expense, we restricted the orientation of the anisotropy axes and the magnetization vectors of all NPs to a single plane containing the magnetic field vector. When calculating the equilibrium free energy of a single NP as a function of its orientation *θ*_*NP*_ in the field $\vec{B}$ (see Figure S8, Equations S7 and S8), one sees that it is minimized when *θ*_*NP*_ =  0 or 180°. Only in this case the mean magnetic moment $\langle {\vec{m}}_{NP}\rangle$ is parallel to the field direction (Note S5 and Figure S9). At low fields, $\langle {\vec{m}}_{NP}\rangle$ can become significantly misaligned from the field, since alignment to the anisotropy axis dominates, while at large fields the misalignment vanishes since alignment with the external field dominates (Figure S9A,B). Free energy barriers for thermally driven flips of the magnetization increase with the applied field and eventually saturate (Figure S9C).

Independent of whether a NP is magnetically anisotropic or not, the force in a field gradient is always given by the product of the absolute value of the mean magnetic moment and the local field gradient, such that it points along the gradient (see Note S5 and Equation S40):

(4) $$\vec{F}=\langle m\left(B\right)\rangle \vec{\nabla }B$$

In the absence of anisotropy (see Note S5), 〈*m*(*B*)〉 is described by a simple Langevin function [29] (Equation S14). For anisotropic NPs, slight deviations from the Langevin behaviour are obtained, depending on the particle orientation. Equation 4 can also be applied to beads consisting of an ensemble of NPs since the mean magnetic moments linearly superimpose.

Within our approach, the torque on a single NP (or even a whole bead) with a given orientation *θ*_*NP*_ in the field can be obtained by differentiating the free energy of magnetization with respect to *θ*_*NP*_ (see Methods). Also, it can be obtained from the mean magnetic moment $\langle {\vec{m}}_{NP}\rangle$ according to (see Note S5 and Equation S55):

(5) $$\vec{\tau }=\langle {\vec{m}}_{NP}\rangle \times \vec{B}$$

At low fields, $\langle {\vec{m}}_{NP}\rangle$ aligns with the anisotropy axis of the NP, such that the torque increases linearly with the field, while at high fields $\langle {\vec{m}}_{NP}\rangle$ aligns more and more with the field such that torque as well as torsional stiffness saturate, the latter at a value of KV (see Note S5 and Figure S10) [11].

To simulate the magnetization and the torsional stiffness of an entire bead, we chose random orientations of a defined number of NPs in a two‐dimensional plane and superimposed their free magnetization energies as a function of the particle angles (Figure 5B,C, Methods). This yielded the total free magnetization energy of the bead as a function of its orientation *θ*_*B*_ with respect to the field. As parameters we used (see Note S5B and Table S2): a volume magnetization of 314 kA/m, NP radii of 6.4 nm for 1‐µm and 5.8 nm for 2.8‐µm beads as well as anisotropy constants of 4.7, 13, and 30 kJ/m^3^. We simulated 71,043 NPs for 1‐µm beads and 685,073 NPs for 2.8‐µm beads to match their experimentally determined total maghemite content. Importantly, the simulations yielded free energy landscapes as a function of the bead orientation that exhibited always two pronounced free energy minima separated by 180°. This indicated the existence of a single well‐defined anisotropy axis for each simulated 1‐µm and 2.8‐µm bead (Figure 5C and Figure S11), as also observed experimentally. Notably, the magnitude of the energy barriers as well as their shapes depended strongly on the bead, i.e. the particular configuration of the NPs (Figure S12).

The free energy minimum of the bead magnetization always corresponded to a perfect alignment of $\langle {\vec{m}}_{B}\left(B\right)\rangle$ with the external field (Figure S11) as previously seen for single NPs. For this orientation, we determined $\langle {\vec{m}}_{B}\left(B\right)\rangle$ as a function of the applied field. Overall, a Langevin function‐like behaviour similar to single NPs was observed (Figure S13). We attribute small deviations from the Langevin function to the anisotropy of the NPs as well as to the restriction of the simulations to two dimensions (Figure S13 and Note S5C). Furthermore, we observed a good agreement between the absolute magnitude of the magnetic moment and experimentally obtained magnetization curves.

### 2.5 Simulations Describe the Torsional Stiffness of Beads in the Field

The barrier height in the free energy profile of a 1 µm bead corresponds to few 100 *k_B_T*, which is much lower than the barrier height of a single NP (up to ∼4 *k_B_T*, see Figure S9C) multiplied by the number of NPs in the bead, yielding a value of ∼280 000 *k_B_T*. Thus, the torsional stiffness of a bead in the field is much lower than one would expect from the total magnetic moment of the bead $\langle {\vec{m}}_{B}\left(B\right)\rangle$.

**FIGURE 6:**
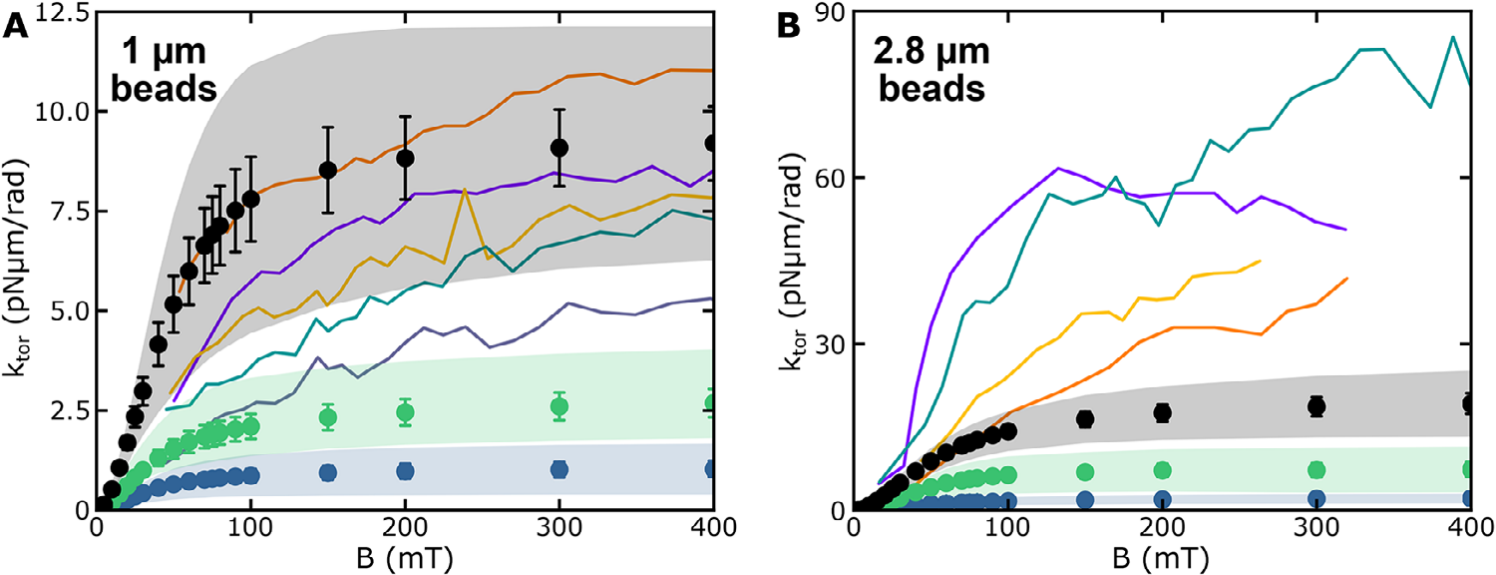
Torsional stiffness of (A) 1‐µm and (B) 2.8‐µm magnetic beads as a function of the applied magnetic field. Black, green and blue circles show the results from simulations using anisotropy constants of 30, 13, and 4.7 kJ/m^3^. Error bars represent standard errors, and shaded areas represent standard deviations. Coloured lines show measured torsional stiffnesses of different (A) MyOne and (B) M270 beads taken from Oene et al. [11].

For quantitative evaluation, we obtained the torsional stiffness directly from the second derivatives of the free energy profiles with respect to *θ*_*B*_ at the equilibrium orientation of the beads. The torsional stiffness started to saturate around a field of 100 mT, and the choice of the anisotropy constant had a large influence on its magnitude (Figure 6). We found a high bead‐to‐bead variation for the torsional stiffness at saturation as seen from the broad standard deviations. Previous measurements by Oene et al. [11]. provided a similar large spread as well as a comparable order of magnitude for the torsional stiffness of single beads (Figure 6). For the 1‐µm beads, we obtained the best agreement for K  =  30 kJ/m^3^ while for K  =  13 kJ/m^3^ the experimental values were slightly and for K  = 4.7 kJ/m^3^ considerably underestimated (Figure 6A, see Discussion and Note S5 for the meaning of the different anisotropy constants). For the 2.8‐µm beads, the simulations provided lower values for the torsional stiffness than the measurements for all tested anisotropies (Figure 6B). Nonetheless, the simulations predicted the correct order of magnitude (see Discussion). Overall, an ensemble of randomly oriented, magnetically anisotropic NPs appears to correctly describe the magnetization as well as the anisotropy of the magnetic beads.

### 2.6 Magnetorelaxometry Measurements Reveal a Broad NP Size Distribution

**FIGURE 7:**
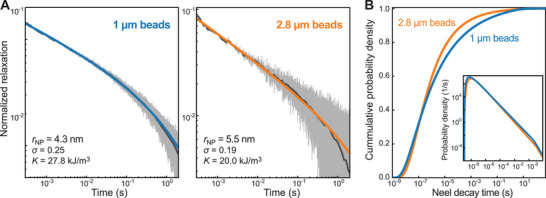
Fluxgate magnetorelaxometry (MRX) measurements. (A) Normalized magnetic B flux after sudden return from 2 mT to zero magnetic field measured for 1‐µm and 2.8‐µm magnetic beads. Shown are the raw signal recorded at 102.4 kHz (light gray), the signal averaged over consecutive time intervals with 20 intervals per decade of equal length on a logarithmic scale (gray) and the fit with the model function (Methods, Equation S20, in blue or orange) yielding the depicted NP parameters. (B) Cumulative distributions of the Néel decay time for the measurements in A calculated from the fit parameters. The inset shows the normalized probability distribution.

So far, we have explored the bead magnetization and its dependence on the NP properties only in equilibrium. The NP size and the anisotropy determine, however, also the dynamics of the magnetization upon field changes. When a magnetic NP rigidly embedded in a matrix becomes magnetized, its magnetization relaxes exponentially with the Néel relaxation time constant $\tau_{N}$ upon removal of the applied field. At zero field the Néel time constant is approximately given by:

(6) $$\tau_{N}=\tau_{0}\;e^{-KV/k_{B}T}$$

where $\tau_{0}$ is a time constant which is typically considered to be 10^−9^ s and KV represents the energy barrier arising from magnetic anisotropy that the magnetic moment must overcome to switch its orientation (as in Equation 3). The distribution of relaxation times thus provides information on the particle volume/size of a given sample. To obtain information on the relaxation dynamics and the particle size distribution, we carried out fluxgate magnetorelaxometry (MRX) measurements on freeze‐dried samples of magnetic beads such that the magnetization could only relax via the Néel mechanism rather than Brownian motion. The samples were magnetized for $t_{\mathrm{mag}}$ = 2 s with a field $B_{0}$ =  2 mT. Subsequently the field was rapidly switched off, and the measurement continued for another 2 s. During the experiment the magnetic flux from the NP magnetization was monitored using a fluxgate magnetometer (Figure S15A), which allowed to resolve the decay of the magnetization starting from ∼300 µs after the field was turned off. The monitored decay exhibited for both bead types a power‐law dependence until ∼10^−1^ s after which the signal dropped more rapidly (Figure 7A). This indicated a broad range of decay times $\tau_{N}$.

In order to estimate the distributions of $\tau_{N}$ and the NP size, we modelled the decay process by assuming a log‐normal distribution of NP radii $r_{\mathrm{NP}}$ being described by the geometric mean of the radii $r_{0}$ and the standard deviation σ of ln $r_{\mathrm{NP}}$. (Equation S13, Methods). We next followed a previous approach [24] and determined the contributions of the different NP sizes to the magnetization at $B_{0}$ (see Methods). Finally, we calculated the decay of the magnetization for this NP ensemble using the expression for the Néel times (Equation 6). Fitting the model to the relaxation data (Figure 7A) revealed significant contributions of Néel times ranging from ∼10^−8^ to ∼1s (Figure 7B). The kink in the Néel time distribution at 1 s that quenches the overall power‐law decay (Figure 7B, inset) was due to the non‐equilibrated initial magnetization of large NPs (Figure S15B). The fit provided mean NP radii of 4.3 and 5.5 nm as well as anisotropy constants of 27.8 and 20.0 kJ m^−3^ for 1‐µm and 2.8‐µm beads, respectively (Figure 7A). The values for the 1‐µm beads are in close agreement with the simulation results. The larger difference for the 2.8‐µm beads maybe due to NP aggregates with higher anisotropies in these beads, which would affect more the torsional stiffness of the beads rather than the measured relaxation times. The obtained values for σ reveal rather broad NP size distributions for both bead types (Figure S15B). Calculated magnetization curves for these NP ensembles were similar to the measured magnetization curves (Figure S15C). Overall, the MRX measurements indicate a significant proportion of NPs that relax in the absence of field on the sub‐second to second range. We note that the upper limit of the obtained decay times was limited by our measurement configuration. At longer time scales, larger NPs with even slower decays would contribute. In agreement with this, we find a residual magnetization of the immobilized beads even at zero field before magnetization in contrast to mobile beads that can relax via Brownian motion (Figure S16).

## 3 Discussion

Here, we characterized the rotational fluctuations of superparamagnetic beads that add to the dynamics of DNA length measurements in magnetic tweezers experiments. Using bead tracking at kHz rates and power spectral density analysis, we could clearly reveal the two different fluctuation modes and assign the high frequency mode to DNA length fluctuations and the low frequency mode to rotational fluctuations. For the latter, the cut‐off frequencies were between 100 and 200 Hz, both for 1‐µm and 2.8‐µm beads, with RMS amplitudes between 10 and 1.4 nm. The values obtained for the torsional stiffness of the beads in the magnetic field were in good agreement with previous measurements [11, 13]. The rotational fluctuations thus considerably perturb the DNA length signal on time scales that are most relevant for magnetic tweezers experiments. A correction of the signal to account for the rotational fluctuations in the time domain is not possible due to the random nature of the fluctuations and the coupling to the DNA length fluctuations. The separation of the two fluctuation modes using PSD analysis becomes generally limited at low forces, where their cut‐off frequencies become similar.

Importantly, the rotational noise contribution decreases with decreasing off‐centre attachment $R_{⊥}$ [13] (Methods, Equation S5). To reduce its influence on DNA length measurements, it is thus important to select magnetic beads with low $R_{⊥}$. Due to its force‐dependence it is also recommended to work at higher forces, which also reduces the DNA fluctuations. Overall, we expect that our careful fluctuation analysis can be used to study rapid biomolecular interactions or conformational changes that only appear as additional noise in DNA length fluctuations.

The rotational fluctuations occur due to the considerable anisotropy of the magnetic beads, which pins the bead orientation along the field lines despite the applied force. To understand the origin of the anisotropy, we modelled single beads as an isotropic ensemble of noninteracting, randomly oriented superparamagnetic NPs. The obtained magnetization free energy landscapes exhibited a high variation between individual beads but always revealed a single well‐pronounced anisotropy axis (Figure S12). This strongly suggests that the anisotropy of the magnetic beads is a finite residue when superpositioning the magnetic moments of the randomly orientated superparamagnetic NPs.

To match the experimental torsional stiffnesses, we had to apply an anisotropy constant of 30 kJ/m^3^ (Figures 2 and 3) being significantly larger than the magnetocrystalline anisotropy of maghemite of ∼5 kJ/m^3^. This suggests that a non‐spherical shape of the NPs, surface effects as well as interactions between the NPs, considerably increase the actual NP anisotropy in agreement with previous measurements [23]. As an estimate of the magnitude of shape effects, we calculated the effective anisotropy constant of a dimer of isotropic NPs with magnetic dipole‐dipole interactions yielding 13 kJ/m^3^ (see Note S5). Larger anisotropy constants can easily arise due to the additional shape anisotropies of the individual NPs of the dimer, size variation and the formation of higher‐order NP clusters with interacting dipoles [18]. While an anisotropy constant of 30 kJ/m^3^ was sufficient to describe the torsional stiffness of 1‐µm beads, an even higher anisotropy, e.g. due to a larger degree of clustering, may be required to explain the torsional stiffness in 2.8‐µm beads (see Figure 6). In line with this, the magnetization curve of 2.8‐µm beads exhibited stronger deviations from a simple Langevin behaviour (between 40 and 60 mT, see Figure S13), which suggests a hindered magnetization against a considerable anisotropy or NP size inhomogeneities. Consistently, in SEM images of 2.8‐µm beads, NP clusters of up to 20 nm could be observed [18].

Interestingly, the average magnetic moment of the beads as well as the torsional stiffness of NPs and the beads exhibit different dependences on the magnetic field (Figures S9, S10, and S13). While 〈*m*(*B*)〉 is Langevin‐like and is half‐saturated at $B_{0}\approx 12-15$ mT (Figure S13), $k_{\mathrm{tor}}\left(B\right)$ of single NPs can be approximated by a rectangular hyperbola [11], such that its asymptotic growth is significantly less steep and half‐saturated at K/M≈ 100 mT (see Note S5 and Figure S10, for K = 30 kJ/m^3^). In contrast, $k_{\mathrm{tor}}\left(B\right)$ of an entire bead exhibits a much steeper initial slope and a rather sudden saturation with half‐saturation at ≈ 40 mT (Figure S14). To understand these differences, it is important to consider that the saturation of $k_{\mathrm{tor}}\left(B\right)$ for individual NPs is governed by the anisotropy constant (instead of $B_{0}$), which sets the curvature of the free energy landscape in vicinity of the energy minimum. For a bead, the free energy landscape is obtained by superpositioning the free energy landscapes of randomly oriented NPs. The curvatures of the individual free energy landscapes around the minimum play thus a minor role, but rather their “overall ripple” set by the energy barriers *G_max_* (*B*) − *G_min_* (*B*) (Figure S9C). When plotting the effective barrier height, it well reproduces the field‐dependence of the torsional spring constant of the beads (Figure S14), such that a plausible explanation for the different field‐dependence of NPs and beads is found.

A large anisotropy constant as suggested from the modelling should strongly affect the (de‐)magnetization dynamics of the magnetic beads. Indeed, the MRX data revealed a very large spread of the decay times ranging from ∼10^−8^ to ∼1s. Quantitative analysis of the relaxation data suggested a wide spread of the effective NP radii ranging from 2.5 to 7.5 nm (Figure S15B) and anisotropy constants of 20–30 kJ/m^3^. This further supports the simulation results and indicates that the maghemite NPs form smaller aggregates in which they magnetically couple. Most likely, even longer relaxation times may be observable when expanding the magnetization time, such that large particles with long relaxation times would become magnetized. When observing such long relaxation times, one may argue that part of the NPs behaves ferrimagnetic rather than superparamagnetic, which in turn may dominate the anisotropy of the beads (Figure S16). We note, however, that in magnetization measurements, hysteresis has so far not been observed [18]. Furthermore, $k_{\mathrm{tor}}$ saturates at large fields in contradiction to a permanent dipole moment with fixed orientation (Equation 5), supporting the idea that the relevant magnetic moments of the NPs can reorient in the field on time scales of the bead fluctuations.

In addition to the fast rotational fluctuations of the beads, we also observed low‐frequency $1/f^{\alpha }$ noise in the PSD measurements with 0.5 ≤ α ≤ 1.5. In contrast to white noise (α  =  0) and Brownian noise (α  =  2), noise with ≈ 1 (pink noise) can have very different sources depending on the particular field [30, 31]. Based on the correlated occurrence of the 1/f‐like noise in axial and lateral direction (Note S4 and Figure S7), we demonstrated that these fluctuations are due to slow direction changes of the anisotropy axis rather than instrument drift. A simple explanation for the drift of the anisotropy axis would be slow random flipping of rather stable magnetic NP moments inside the bead (Figure 5B). We note, however, that for the obtained anisotropy constant, the NPs would possess only a single preferred magnetization direction rather than two metastable directions for fields above 100 mT (Figure S9A). Given magnetic fields of up to 500 mT in our experiments, random domain flips may only be due to highly anisotropically shaped iron oxide NPs with correspondingly higher anisotropy constants. TEM images reveal the presence of larger NP aggregates in the beads, supporting this idea [18]. Alternatively, changes in the magnetization direction of individual NPs may also be due to NPs that are not rigidly attached in the bead matrix but rather free to adopt more than a single position.

The extent to which the 1/f‐like fluctuations of the anisotropy axis are observed in the axial direction increases with increasing off‐center attachment (Figure S7). Furthermore, the (anti‐) correlation between the axial and the lateral low‐frequency fluctuations can be employed to partially correct the axial fluctuations using the lateral fluctuations (Figure S7). This considerably increases the long‐term stability of the DNA length measurements and thus the spatio‐temporal resolution on the 1‐nm scale. This correction is, however, only possible for low‐frequency noise occurring at lower frequencies than the lateral position fluctuations of the beads. Altogether, we recommend a careful selection of magnetic beads with small off‐center attachment, for which both the high‐frequency and the low‐frequency rotational fluctuations appear at greatly reduced extent along the axial direction.

## Author Contributions

R.S. designed the research. S.B., F.W. and A.L. carried out the experiments. S.B. and F.W. designed and produced the DNA substrate. S.B., F.W., A.L. and R.S. analyzed the data. S.B. and R.S. designed and wrote the simulations while D.J.K. helped improve the performance. S.B., R.S. and A.L. interpreted the results. S.B., A.L. and R.S. wrote the manuscript. D.J.K. and F.W. provided comments to the manuscript.

## Conflicts of Interest

The authors declare no conflicts of interest.

## Supporting information




**Supporting File**: smll71982‐sup‐0001‐SuppMat.pdf

## Data Availability

The data that support the findings of this study are available from the corresponding author upon reasonable request.
