# Supplementary material for: Origin of the Magnetization Anisotropy of Superparamagnetic Beads
Source: Small. 2026 Jan 8;22(9):e08156. doi: 10.1002/smll.202508156 (PMC12895231; doi:10.1002/smll.202508156)
Supplement: Supplementary file 1 — Supporting File: smll71982‐sup‐0001‐SuppMat.pdf [file SMLL-22-e08156-s001.pdf]

Supporting Information

Origin of the magnetization anisotropy of superparamagnetic beads

Sebastian Belau[1], Fabian Welzel[1], Dominik J. Kauert[1], Aidin Lak[2], and Ralf Seidel[1]

[1]Peter Debye Institute for Soft Matter Physics, University of Leipzig, Leipzig, Saxony, 04103 Germany

[2]Institute for Electrical Measurement Science and Fundamental Electrical Engineering and Laboratory for Emerging Nanometrology (LENA), TU Braunschweig, Hans-Sommer-Str. 66, 38106 Braunschweig, Germany

Methods

DNA substrate preparation

The DNA-construct for the magnetic tweezers experiments was prepared from two linear PCR fragments with lengths of 5326 bp and 4229 bp that were fabricated using PCR primers integrating desired restriction sites (see SI Note S1, Table S1). The longer fragment was digested with the restriction enzymes Nt.BbvCI and PspOMI while the shorter fragment was digested with Nt.BbvCI and XhoI. Digestion with the nicking enzyme Nt.BbvCI yielded mutually complementary 9 nt 3’ overhangs on the two fragments to allow their ligation. For attachment to the flow-cell surface and magnetic beads, 1136-bp biotin- and digoxigenin-modified DNA handles were produced by PCR from plasmid pBluescript II SK+ (see Table S1) using either biotin- or digoxigenin-modified dUTPs (21). The PCR-products were then digested with PspOMI (biotin-handle) or XhoI (digoxigenin-handle), with their respective sites located at the centre of the PCR fragment, yielding two handle molecules with one sticky end per PCR molecule. The four different fragments were then ligated together in a single reaction producing a 9546-bp dsDNA construct (about 3.1 µm contour length) carrying on one end a biotin- and on the other end a digoxigenin-fragment. The final construct was purified from an agarose gel, while avoiding exposure to ethidium bromide and UV-illumination[1]. For the experiments in Fig. S7 a 2.1 kbp construct was prepared from plasmid pUC19 as previously described[2] using the modified DNA handles described above.

Table S1: List of primers used for creating the DNA construct and handles via PCR.

Name		Sequence
Fwd	construct	5'-G G C G C C G C C T C A G C C G C A G G A A A G A A C A T G T G A G-3'
Rev 4229	construct	5'-T T A T C G A T A C C G T C G A C C T C G A G C A G C A A C C G C A-3'
Rev 5326	construct	5'-A A A C A G G C T C T G C G G G C C C G G C G T-3'
pBlueFor	handles	5'-G A C C G A G A T A G G G T T G A G T G-3'
pBlueRev2	handles	5'-T T T G T G A T G C T C G T C A G G G G-3'

biotin- and digoxigenin-handles were created from the plasmid pBlueScript II SK+
DNA construct was created from the modified plasmid pBluescript+1+2+4 (sequence can be obtained upon request)
coloured sequences mark the restriction sites for Nt.BbvCI (red), XhoI (blue) and PspOMI (green) respectively

Flow cell preparation

Flow cells for magnetic tweezers experiments were assembled using two glass coverslips (Menzel Gläser). The top coverslip contained two 2 mm holes to allow sample exchange. The bottom coverslip was spin-coated with a 1% polystyrene solution at 6000 rpm. The two coverslips were glued together using a Parafilm spacer (Bemis, Oshkosh, USA) containing a single flow channel and heating to 120°C on a hotplate. Prior to measurements, we added 3 µm carboxylated polystyrene beads serving as reference during bead tracking (Invitrogen) suspended in experiment buffer (10 mM Tris, 150 mM NaCl and 1 mM EDTA at pH 8) and let them incubate over night to allow adherence to the bottom cover slip. Afterwards the cell was incubated with 50 mg/ml antidigoxigenin in phosphate-buffered saline for 1 h followed by incubation with bovine serum albumin for at least 1 h, to prevent unspecific binding to the surface.

Magnetic tweezers experiments

Experiments were conducted using a self-built high-resolution magnetic tweezers setup[3, 4] (see Fig. 1A) which was based on a compact self-built inverted microscope with a 100x oil immersion objective (NA 1.25, Olympus, Center Valley, PA). To provide sufficient illumination of the beads at tracking frequencies of 2.8 kHz, a fibre-coupled mercury arc lamp (LSB610, LOT-Oriel, Darmstadt, Germany) and a 550/100 nm band pass filter (AHF Analysentechnik, Tübingen, Germany) were used.

The external magnetic field was generated by a pair of NdFeB permanent magnets with vertical magnetization and mutual anti-parallel alignment (see Fig. 1A). Each magnet consisted of three magnets (1xQ-05-05-03-N52N, 2xQ-05-04-01-G, Supermagnete, Gottmadingen, Germany) stacked on top of each other to create a geometry that allows for better illumination. The magnet pair was mounted onto a motorized stage to allow axial/vertical magnet translations as well as magnet rotations. A high-speed CMOS camera (EoSens CL MC1362, Mikrotron, Unterschleißheim, Germany) was used for real-time bead imaging. The images were transferred to a computer using an image acquisition card (NI PCIe 1429, National Instruments, Austin, Texas) and analysed using a fast GPU (GeForce GTX 480, Nvidia, Santa Clara, CA) as described previously[4].

Prior experiments, the flow cell containing the polystyrene reference beads was mounted onto a nanopositioning stage (P-517.3 CD, Physik Instrumente, Karlsruhe, Germany). The DNA was bound to streptavidin-coated magnetic beads suspended in the experiment buffer and flushed into the flow cell to allow attachment to the surface. After 2 to 3 min the channel was washed with the same buffer to remove unbound material. Subsequently, the magnets were lowered to apply force to DNA-tethered magnetic beads. As magnetic beads, we employed either MyOne or M-280 Dynabeads (Invitrogen) with a diameter of 1 µm and 2.8 µm respectively.

All experiments were carried out at 20.5°C. The tight binding of the reference beads to the polystyrene-coated surface was verified using particle tracking and PSD analysis. During the experiments the position of a DNA tethered bead with tracked simultaneously with a reference bead. The DNA length was calculated from the axial position difference of the two beads, which effectively removed drift of the microscope stage. The forces exerted on the magnetic beads were calibrated by analysing their lateral fluctuations as described before[5] .

PSD analysis and fitting

PSD spectra were calculated from bead position trajectories of 900 s length that were divided into $n = 40$ or 250 equally long segments for M280 and MyOne magnetic beads, respectively.

To analyze the experimentally obtained PSDs, we described our DNA tethered beads as a system of two coupled overdamped oscillators. For small displacements, the entropic elasticity of DNA is linear. Its spring constant $k_{DNA} = dF_{DNA}/dz_{DNA}$ is given by the derivative of the DNA force-extension relation with respect to the DNA extension z_{DNA} at the given force as given by the worm-like-chain (WLC) model[6, 7]. The attached magnetic bead with radius R causes a viscous drag force for translations along the z-direction with drag coefficient $\gamma_{trans} = 6\pi\eta R$. For pure DNA length fluctuations, the PSD of the axial bead positions is provided by a simple Lorentzian function[8] according to:

$$PSD_{Z,DNA}(f) = \frac{4k_B T\gamma_{trans}}{k_{DNA}^2}\frac{1}{1+(f/f_{DNA})^2} \tag{S1}$$

where k_B is the Boltzmann constant, T the temperature and $f_{DNA} = k_{DNA}/2\pi\gamma_{trans}$ the characteristic cut-off frequency. The total mean-square displacement over the entire frequency range is given by $\langle z_{DNA}^2\rangle = k_B T/k_{DNA}$ in agreement with the equipartition theorem applied to a harmonic oscillator.

Rotational fluctuations of the bead around its preferred alignment (x-axis, Fig. 1C) cause, in first approximation, a back-driving torque $\Gamma_{tor} = -k_{tor}\delta\varphi$ for an angular displacement $\delta\varphi$, with k_{tor} denoting the torsional stiffness of the bead in the field. Additionally, rotational displacements of the bead are counteracted by the rotational viscous drag $\Gamma_D = -\gamma_{tor}\delta\dot{\varphi}$, with $\gamma_{tor} = 8\pi\eta R^3$ being the rotational drag coefficient. In presence of an off-centre attachment of $R_\perp$, the rotational displacement changes the height of the DNA attachment point by δz_{rot} (Fig. 1C), such that it couples to the linear DNA length fluctuation of magnitude δz_{DNA}. The distance of the bead center from flow-cell surface is given by $z = z_{DNA} + z_{rot}$. At the DNA attachment point, the two torque components translate into linear forces $F_{tor} = -k_{tor}/R_\perp^2\delta z_{rot}$ and $F_{D,tor} = -\gamma_{tor}/R_\perp^2\dot{z}_{rot}$ along the DNA axis[3]. Bead translations are driven by the DNA stretching force, the translational drag and the corresponding random force $F_{trans}(t)$. Bead rotations are driven by the rotational displacement torque, the DNA stretching force, the rotational drag as well as the corresponding random force $F_{rot}(t)$. The coupled bead translation and rotation can thus be comprised in a pair of coupled Langevin equations[3]:

$$\begin{aligned} &-\gamma_{trans}\dot{z} - k_{DNA}z_{DNA} = F_{trans}(t) \\ &-\gamma_{rot}\dot{z}_{rot} - k_{rot}\delta z_{rot} + k_{DNA}z_{DNA} = F_{rot}(t) \end{aligned} \tag{S2}$$

with $\gamma_{rot} = \gamma_{tor}/R_\perp^2$ and $k_{rot} = k_{tor}/R_\perp^2$ being the effective linear contributions from rotational bead fluctuations. An analytical solution of this set of Langevin equations for the PSD of the bead position fluctuations has been derived before[3] and is provided by a sum of two Lorentzians:

$$\begin{aligned} PSD_z^{coupl}(f) = \frac{4k_B T}{1+D^2\gamma_{trans}\gamma_{rot}}\Bigg\{&\frac{\gamma_{rot}D^2}{\left(2\pi f_+^{coupl}\right)^2+(2\pi f)^2} \\ &+\frac{1}{\gamma_{trans}\left[\left(2\pi f_-^{coupl}\right)^2+(2\pi f)^2\right]}\Bigg\}. \end{aligned} \tag{S3}$$

$f_\pm^{coupl}$ are the cut-off frequencies of the two fluctuation modes of the coupled system, $D = (2\pi f_-^{coupl} - (k_{DNA}+k_{rot})/\gamma_{rot})/k_{DNA}$, k_B the Boltzmann constant and T the temperature. The cut-off frequencies $f_\pm^{coupl}$ are given by[3]:

$$f_\pm^{coupl} = \frac{k_{DNA}+k_{rot}}{4\pi\gamma_{rot}} + \frac{k_{DNA}}{4\pi\gamma_{trans}} \pm \frac{1}{4\pi}\sqrt{\left(\frac{k_{DNA}+k_{rot}}{\gamma_{rot}}+\frac{k_{DNA}}{\gamma_{trans}}\right)^2 - \frac{4k_{DNA}k_{rot}}{\gamma_{trans}\gamma_{rot}}} \tag{S4}$$

The off-center attachment $R_\perp$ determines the coupling strength between the two types of oscillations. When using the cut-off frequencies $f_{DNA} = k_{DNA}/2\pi\gamma_{trans}$ and $f_{rot} = k_{rot}/2\pi\gamma_{rot}$ of the individual oscillators in absence of coupling, Eq. S4 can be rewritten (see SI Note 1). For weak coupling between the two oscillations, i.e. $R_\perp < R$, and sufficiently distinct cuttoff frequencies of the single, uncoupled oscillators, i.e. $f_{rot} \ll f_{DNA}$, the characteristic frequencies of the coupled system can be approximated (see Note S1, Eq. S25). This reveals that, f_+^{coupl} is dominated by the DNA length fluctuations with f_+^{coupl} slowly increasing from f_{DNA} with increasing coupling, while f_-^{coupl} is dominated by the rotational fluctuations of the bead with f_-^{coupl} slowly decreasing from f_{rot} with increasing coupling (see Note S1, Fig. S1).
Independent of the coupling strength, the total mean-square displacement of the bead fluctuations is always given by the mean-square displacements of the individual oscillators:

$$\langle z^2 \rangle = \langle z_{DNA}^2 \rangle + \langle z_{rot}^2 \rangle = k_B T/k_{DNA} + k_B T/k_{rot} \tag{S5}$$

To account for low frequency noise, not being captured by Eq. S3, an additional $1/f$-like term $PSD_{1/f}$ was added:

$$PSD_{1/f} = \frac{A}{f^\alpha} \tag{S6}$$

A is the amplitude of the low frequency noise and $0.5 < \alpha < 2$ the power of the decay.
The model PSD functions were corrected for low-pass filtering and aliasing from the camera detection as described in the SI Note S2[5, 9, 10]. Direct weighted fitting of PSD data with the corrected model functions was carried out as described before, using PSD_{exp}^{-2}/n as weights, with n being the number of PSD sub-trajectories. In practice, standard least-mean-square fitting of the reciprocal experimental data PSD_{exp}^{-1} with the reciprocal model PSD was carried out. Correction of an associated bias during PSD fitting was accomplished by multiplication of the best fit function with $n/(n+1)$.
The off-center attachment $R_\perp$ was determined for each bead and measurement condition by slowly rotating the magnet configuration and determining the radius of the resulting circular bead trajectory in the xy-plane[3] which was additionally corrected for off-axis pulling (SI Note S3, Fig. S3). For the 1-µm beads an accurate experimental determination of the off-centre attachment was not possible, since the relative contribution of the off-axis pulling was too large (see Fig. S6). $R_\perp$ was therefore calculated from the axial component of the rotational drag coefficient γ_{rot} obtained in the PSD analysis (see below) and the expected rotational drag coefficient of a bead with radius R using $R_\perp^2 = 8\pi\eta R^3/\gamma_{rot}$. Determined values of $R_\perp$ were between 0.045 and 0.12 µm as well as 0.35 and 0.75 µm for 1-µm and 2.8-µm beads, respectively (Fig. S4).

Simulations of the bead magnetization

Simulations of the magnetization and the anisotropy of magnetic beads were performed using a Python (3.8.5) script. Beads were simulated as a homogeneous ensemble of anisotropic superparamagnetic NPs with random orientation. Anisotropy axes and magnetic moment vectors were constraint to a plane containing the field vector $\vec{B}$. Mutal dipole-dipole interactions between the NPs were neglected. As parameters we used the volume magnetization $M = 314\ kA/m$, the anisotropy constant C with values of 30 kJ/m³, 13 kJ/m³ and 4.7 kJ/m³ and NP radii of 6.4 nm for 1-µm (MyOne) and 5.8 nm for 2.8-µm (M280) beads, respectively (see Results and SI note S4 for the choice of this parametrization). The number of simulated NPs was 71,043 for the 1-µm and 685,073 for the 2.8-µm beads, respectively. For a given NP

we calculated its mean free energy as function of its orientation θ_{NP} with respect to the field according to:

$$G(B,\theta_{NP}) = -k_B T \ln(Z) \quad \text{(S7)}$$

with k_B being the Boltzmann constant, T the temperature and Z the partition function, which is obtained by integrating over all possible orientations θ_2 of the magnetic moment $\vec{m}$:

$$Z(B,\theta_{NP}) = \int_0^{2\pi} e^{-\frac{U(B,\theta_{NP},\theta_2)}{k_B T}} d\theta_2 \quad \text{(S8)}$$

with $U(B,\theta_{NP},\theta_2)$ being the potential energy of the NP dipole inside the field given by Eq. 3 (main text). The mean magnetic moment of the NP parallel to the field is then given by:

$$\langle m_{par}\rangle(B,\theta_{NP}) = \frac{1}{Z}\int_0^{2\pi} m\cos\theta_2 \; e^{-\frac{U}{k_B T}} d\theta_2 \quad \text{(S9)}$$

while its perpendicular component is given by:

$$\langle m_{perp}\rangle(B,\theta_{NP}) = \frac{1}{Z}\int_0^{2\pi} m\sin\theta_2 \, e^{-\frac{U}{k_B T}} \sin\theta_2 \, d\theta_2 \quad \text{(S10)}$$

with $m = MV$ being the magnitude of the magnetic moment, where $V = 4/3\,\pi R^3$ is the NP volume. The integration was done numerically. To obtain the free energy $G(B,\theta_B)$ as well as the components of the magnetic moment $\langle m_{par/perp}\rangle(B,\theta_B)$ as function of the bead orientation θ_B for an entire bead, all free energies of the individual NPs and their magnetic moments were summed up. We note that for a given field B we had to calculate $G(B,\theta_{NP})$ and $\langle m_{par/perp}\rangle(B,\theta_{NP})$ only for a single NP, since for other NP orientations these functions could be simply shifted by the respective angular difference, greatly simplifying the simulations.
The torque that acts on a NP or bead to align its axis with the external field, was obtained from the negative derivative of $G(B,\theta_{NP/Bead})$ with respect to θ_{NP} or θ_{Bead}:

$$\Gamma(B,\theta_{NP}) = -\frac{\partial G(B,\theta_{NP})}{\partial \theta_{NP}} \quad \text{(S11)}$$

The torsional stiffness considering small displacements from a free energy minimum was thus given by the second derivative, i.e. the curvature of the simulated free energy landscape at the minimum:

$$k_{tor} = -\left(\frac{\partial \Gamma(B,\theta_{NP})}{\partial \theta_{NP}}\right)_{min} = \left(\frac{\partial^2 G(B,\theta_{NP})}{\partial \theta_{NP}^2}\right)_{min} \quad \text{(S12)}$$

Fluxgate magnetorelaxometry measurements and analysis

MRX measurements were performed on suspension and freeze-dried samples of magnetic beads. The freeze-dried samples were prepared by mixing 150 µl of an aqueous suspension of the beads with a previously freeze-dried mannitol matrix and freeze drying it overnight. The measurements were performed on a custom-made fluxgate-based magnetorelaxometer in a magnetically shielded chamber. The sample is first magnetized at 2 mT for 2 s and then the magnetization decay is recorded for 2 s.
For analyzing the decay of the magnetization, we subtracted the initial magnetic flux before magnetization and normalized the relaxation trajectories by the saturation magnetization upon applying the magnetization field B_0 of 2 mT. We also removed a delay of 2 ms from the relaxation time to account for a delayed response of the flux magnetometer. We next modelled

the relaxation process as described[11] by assuming a log-normal distribution of nanoparticle radii:

$$p(r) = \frac{1}{\sqrt{2\pi}\,\sigma_r\, r} e^{-(\ln r - \ln r_0)^2/2\sigma_r)^2} \tag{S13}$$

where r_0 is the geometric mean of the distribution and σ_r the standard deviation of $\ln r$. The NP volumes are then also log-normal distributed with geometric mean $V_0 = 4/3\,\pi r_0^3$ and $\sigma_V = 3\sigma_r$. When magnetizing the NPs with a field B_0 only a partial radius-dependent equilibrium magnetization is achieved. Its fraction is given by the Langevin equation:

$$L(B,V) = \coth(\,BMV/k_B T) \,-k_B T/BMV \tag{S14}$$

M is the volume magnetization. Particularly small NPs with small magnetic moments become only little magnetized. In addition, larger NPs with large Néel relaxation times partially kinetically fail to become magnetized during the time window t_{mag}. The fraction of NPs that become magnetized within t_{mag} can be calculated as described[11]. Let:

$$\begin{aligned} B_k &= \frac{K}{M}, \\ K_{mod} &= (K - 5\cdot 10^3)/3\cdot 10^3 \\ \sigma_{mod} &= \frac{\sigma_r - 0.35}{0.15} \\ F &= 2.77 + 0.17\,K_{mod} - 0.03\,K_{mod}^2 - 0.015\,\sigma_{mod} K_{mod} \end{aligned} \tag{S15}$$

Then the Néel relaxation time of the magnetization in presence of an external field is given by:

$$\tau_{NH}(B,V) = \tau_0 \exp\left\{\frac{MV}{k_B T}\left[1 - F\frac{B_0}{B_k} + \left(\frac{B}{B_k}\right)^2\right]\right\} \tag{S17}$$

with $\tau_0 = 10^{-9}\,s$. The fraction of particles that undergo a Néel relaxation within t_{mag} is then:

$$F_{NH}(t_{mag}, B, V) = 1 - \exp(-t_{mag}/\tau_{NH}(B,V)) \tag{S18}$$

The contribution of a given particle radius to the magnetization at B_0 is thus:

$$p_{mag}(r, t_{mag}, B_0) = \frac{1}{Norm} MV\, p(r)\, L(B_0, V)\, F_{NH}(t_{mag}, B_0, V) \tag{S19}$$

with $V = 4/3\,\pi r^3$ and Norm being a numerically obtained normalization constant to ensure that $p_{mag}(r, t_{mag}, B_0)$ is a normalized probability distribution. The normalized model relaxation curve is then obtained by superimposing the relaxation curves of the different NP radii using $p_{mag}(r, t_{mag}, B_0)$ as weights:

$$m_{norm}(t) = \int_0^\infty \exp\left(-\frac{t}{\tau_N(r)}\right) p_{mag}(r, t_{mag}, B_0)\, dr \tag{S20}$$

where the integration was done numerically. $\tau_N(r)$ are the Néel relaxation times in absence of an external field given by:

$$\tau_N(r) = \tau_0 e^{-KV/k_B T} \tag{S21}$$

Eq. S20 was fit to the measured relaxation curves in order to obtain best fit values for r_0, σ_r and K. To reduce the numeric effort for the fitting, the experimental curves were downsampled by averaging them over consecutive time intervals of equal length on a logarithmic scale with 20 intervals per decade (dark gray curves in Fig. 7A).

Note S1 – Approximation of the cut-off frequencies of the coupled system

Using the expressions for the cut-off frequencies $f_{DNA} = k_{DNA}/2\pi\gamma_{trans}$ and $f_{rot} = k_{rot}/2\pi\gamma_{rot}$ of the individual oscillators in absence of coupling, the equation for the cut-off frequencies of the coupled system (Methods, Eq. S4) can be transformed to:

$$f_{\pm}^{coupl} = \frac{f_{DNA}}{2}\left(\frac{\gamma_{trans}}{\gamma_{rot}} + 1\right) + \frac{f_{rot}}{2} \pm \sqrt{\frac{1}{4}\left(f_{DNA}\left(\frac{\gamma_{trans}}{\gamma_{rot}} + 1\right) + f_{rot}\right)^2 - f_{DNA} f_{rot}} \tag{S22}$$

where the ratio between the drag coefficients can be expressed as:

$$\frac{\gamma_{trans}}{\gamma_{rot}} = \frac{6\pi\eta R}{8\pi\eta R^3/R_\perp^2} = \frac{6R_\perp^2}{8R^2} < 1 \tag{S23}$$

Plotting $f_{\pm}^{coupl}$ as function of $R_\perp/R$ provides that the cut-off frequencies of the coupled system increasingly deviate from f_{DNA} and $f_{rot}$with increasing relative off-center attachment $R_\perp/R$, which thus represents the coupling strength (see Fig. S1). Using a first order Taylor expression of $f_{\pm}^{coupl}$ one obtains in the limit of small $R_\perp/R$:

$$f_{\pm}^{coupl} \approx \begin{cases} f_{DNA} + f_{DNA}\dfrac{f_{DNA}}{f_{DNA} - f_{rot}}\dfrac{6R_\perp^2}{8R^2} \\ f_{rot} - f_{rot}\dfrac{f_{DNA}}{f_{DNA} - f_{rot}}\dfrac{6R_\perp^2}{8R^2} \end{cases} \tag{S24}$$

For $f_{rot} \ll f_{DNA}$ this can be further simplified to:

$$f_{\pm}^{coupl} \approx \begin{cases} f_{DNA}\left(1 + \dfrac{6R_\perp^2}{8R^2}\right) \\ f_{rot}\left(1 - \dfrac{6R_\perp^2}{8R^2}\right) \end{cases} \tag{S25}$$

which provides a good approximation for the exact expression for the relevant range of $R_\perp$ (Fig. S1). Even for very slow rotational fluctuations compared to DNA length fluctuations, the measured cut-off frequencies stay highly dependent on $R_\perp$.

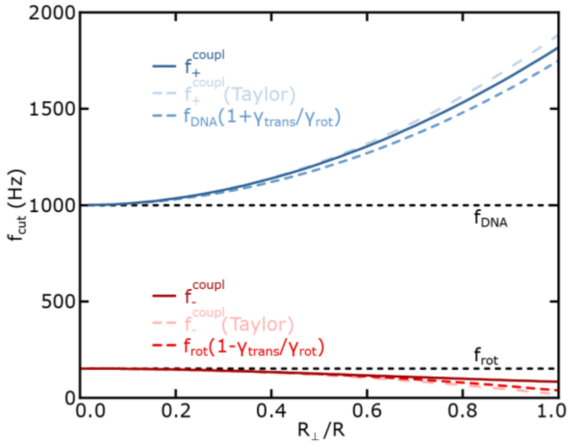

Figure S1: Cut-off frequencies f_+^{coupl} (dark blue solid line) and f_-^{coupl} (dark red solid line) of the coupled oscillator system as function of the coupling strength $R_\perp/R$. Black dashed lines represent the cut-off frequencies of the uncoupled oscillators. The light blue/red dashed lines show the Taylor-approximation and a further simplification for the case $f_{rot} \ll f_{DNA}$.

Note S2 – Correcting the PSD formula for filtering by the camera acquisition

The PSD for the axial position fluctuations of the magnetic bead is given by the sum of the $1/f$-like noise (Eq. S6) as well as the coupled DNA-length and rotational fluctuations:

$$PSD_z(f) = PSD_z^{coupl} + PSD_{1/f} \quad \text{(S26)}$$

The camera detection alters, however, the PSD of the DNA-tethered beads due to low-pass filtering and aliasing[3, 12, 13]. The model function for the bead fluctuations used for the PSD fitting were thus corrected to include the camera filtering according to:

$$PSD_z^{corr}(f) = \sum_{n=-\infty}^{\infty} PSD_z(|f + nf_s|) \frac{sin^2(\pi\tau_e|f + nf_s|)}{(\pi\tau_e|f + nf_s|)^2} \quad \text{(S27)}$$

where $f_s = 2.8\ kHz$ is the camera sampling frequency and $\tau_e = 1/f_s = 0.35\ ms$ the exposure time of the camera. The term for $n = 0$ represents the PSD that is only corrected for low-pass filtering, while higher order terms are attributed to frequencies higher than $f_s/2$ that contribute to the spectrum (alias correction). For $f_s = 1/\tau_e$ only the term for $n = -1$ is needed to sufficiently correct for aliasing[13], as also performed in this work. Fig. S2 shows the best fit of an experimental PSD using the detection-corrected model functions of a single oscillator, two coupled oscillators and two coupled oscillators with additional 1/f-like noise.

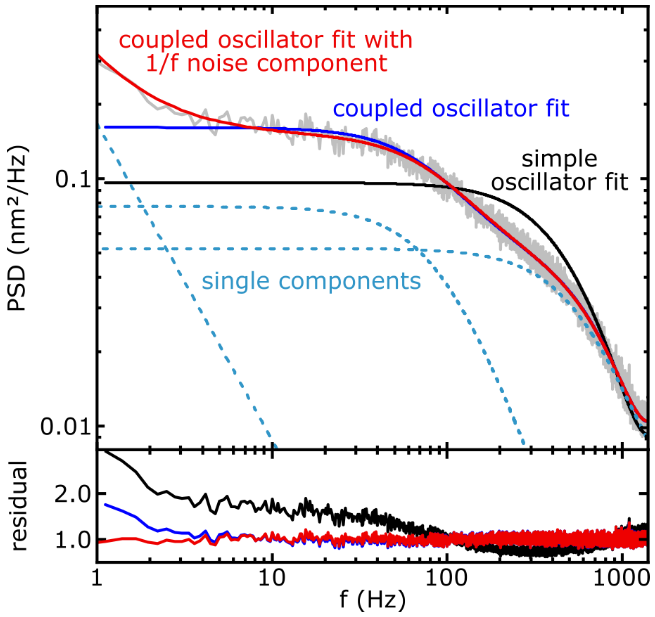

Figure S2: PSD of the time trajectory shown in Fig. 1B main text (grey) fitted with a single Lorentzian (black, corresponding to a single overdamped oscillator), a double Lorentzian (blue, corresponding to two coupled overdamped oscillators) and a double Lorentzian with additional 1/f-like noise (red). The experimental PSDs normalized by division with the model PSD are shown in the same colours below to illustrate the relative deviations. The light blue dashed lines represent the single components of the double Lorentzian fit including 1/f-like noise. All model functions were corrected for alterations by the camera detection.

Note S3 - Effective hydrodynamic radii and off-center attachment correction

The torsional drag coefficient γ_{tor} and the torsional stiffness k_{tor} estimated from PSD fitting depends strongly on the determined off-center attachment of the DNA on the magnetic bead $R_{\perp}$, which arrises from the pinning of the bead orienbtation along the magnetic field. A magnetic force that has a small angular deviation θ_{tilt} from the axial direction (i.e. the rotation axis of the magnets) can cause a significant error during the determination of $R_{\perp}$ by bead rotations due to an additional radial displacement R_{tilt} of the DNA attachment point on the bead (see Fig. S3). The apparent off-center attachment $R_{\perp}^{app}$ as measured by magnet rotations (see Methods) is thus given by:

$$R_{\perp}^{app} = (R_{tilt} + R_{\perp}) = R_{\perp} \pm L_{DNA} \sin\theta_{tilt} \qquad \text{(S28)}$$

where L_{DNA} is the DNA length, θ_{tilt} the tilt angle with respect to the axial direction and $R_{\perp}$ the real off-center attachment on the bead. By determining θ_{tilt} from the tracked 3D trajectory of the bead, $R_{\perp}$ was corrected for non-axial pulling. Note that the tilt term in the equation above can be positive or negative depending on whether the tilting goes along or opposite to the direction of the off-center attachment.

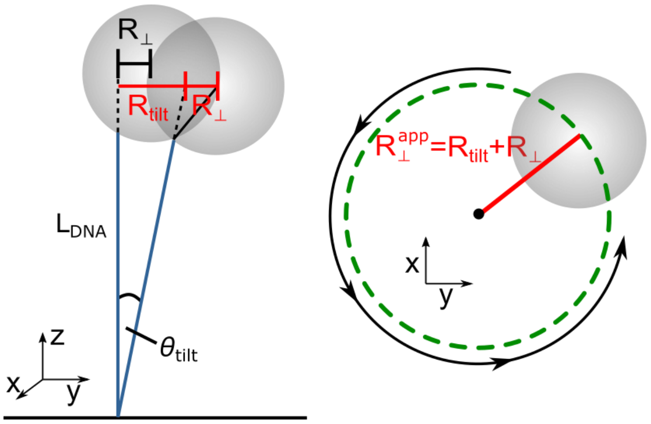

Figure S3: Schematic for the correction of the measured apparent off-center attachment $R_{\perp}^{app}$. A possible tilt of the force with respect to the magnet rotation axis adds (or subtracts, depending on the direction of the tilt) a tilt radius R_{tilt}.to the actual off-center attachment $R_{\perp}$.(Left) Side view of the DNA-tethered bead to illustrate the tilted pulling force. (Right) Top view on the configuration including the bead trajectory during magnet rotation.

The measured off-center attachments for the 1-µm and 2.8-µm beads can be seen in Fig. S4 for the applied forces. A general decrease of $R_{\perp}$ for increasing force can be observed in the case of 2.8-µm beads, as has been reported previously[3], which arises due to the rotational displacement of the bead out of its preferred orientation at higher forces.

Figs. S5 and S6 show the effective hydrodynamic radii as function on force. As mentioned in the main text, they were calculated from the obtained values for γ_{trans} as well as γ_{tor} respectively. An alternative method to determine the radii is shown in inset of Fig. S6 by plotting $\gamma_{rot}(R_{\perp})$ and fitting the data with $\gamma_{rot} = 8\pi\eta R^3/R_{\perp}^2$ ($\eta = 10^{-3} kgs^{-1}m^{-1}$). This method yields an effective hydrodynamic radius of 1.54 ± 0.62 µm for 2.8-µm beads (for forces of 12.5 pN and above). In Fig. S5 and S6 we can see that for lower forces the hydrodynamic radius is more accurate when determined from γ_{trans}, while at higher forces γ_{tor} provides

more accurate radii. The reason for this is likely that at low forces the DNA bending fluctuations are dominant in the power spectrum and at higher forces the bead rotation fluctuations dominate, making it easier to fit that respective mode.

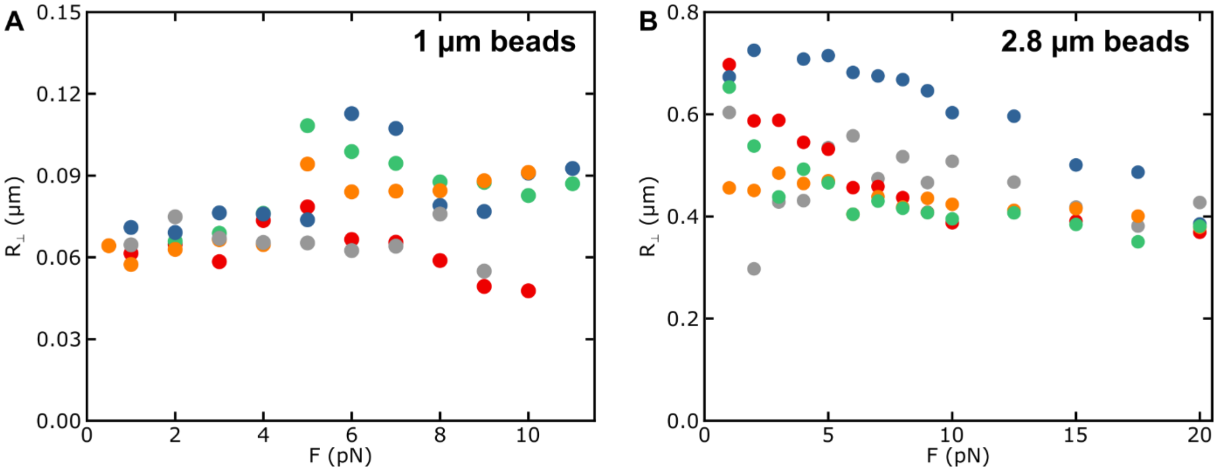

Figure S4: Off-center attachment $R_\perp$ of DNA-tethered 1-µm magnetic beads (A) and 2.8-µm magnetic beads (B) at different stretching forces. Different colours stand for different beads and match the colour scheme of Fig. 2 D, E and Fig. 3 D, E respectively.

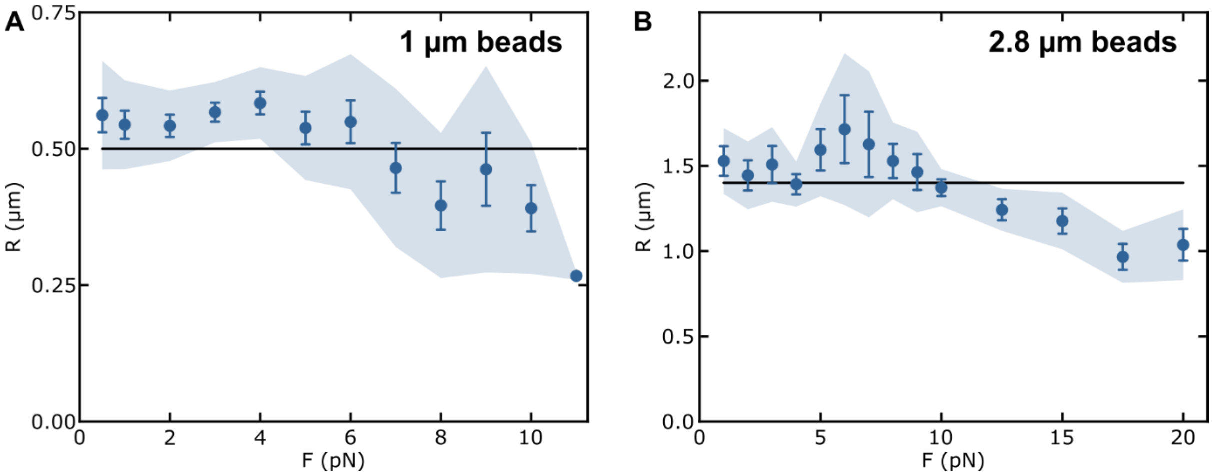

Figure S5: Effective hydrodynamic radii R of 1-µm magnetic beads (A) and 2.8-µm magnetic beads (B) determined from the translational drag coefficient $\gamma_{trans} = 6\pi\eta R$. The error bars represent the standard errors of the mean and the shaded area the standard deviation from 10 beads (1-µm) and 5 beads (2.8-µm). Block solid lines indicate the nominal radii. For 2.8-µm beads a correction for the proximity of the surface according was applied[14] ($\eta = 10^{-3} kgs^{-1}m^{-1}$).

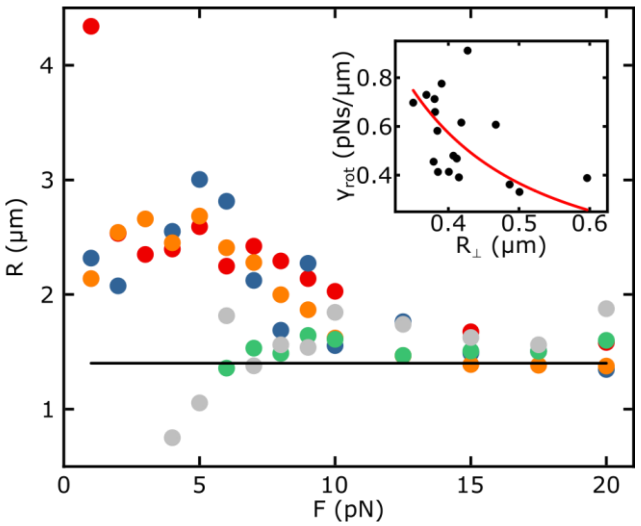

Figure S6: Effective hydrodynamic radii R of the 2.8-µm magnetic beads obtained at different forces from the rotational drag coefficient $\gamma_{tor} = 8\pi\eta R^3$. Different colours represent the results for different particles. The black line marks the nominal bead radius of 1.4 µm. For forces above 12.5 pN an average bead radius of 1.55 ± 0.15 µm was obtained. At these forces the two fluctuation modes have well separated cut-off frequencies which supports a higher fitting accuracy. The inset shows the axial component γ_{rot} of the rotational drag coefficient as obtained from the PSD fitting as function of the off-center attachment $R_\perp$ (black solid circles). The red line as a fit to the data according to the expected relation $\gamma_{rot} = 8\pi\eta R^3/R_\perp^2$ ($\eta = 10^{-3} kg s^{-1} m^{-1}$) for which we obtained a radius of 1.5 ± 0.6 µm.

Note S4 – Slow orientation fluctuations of the bead anisotropy axis

To investigate in more detail the origin of the 1/f-like noise of the axial bead position, we recorded long position trajectories using 2.1 kbp (0.70 µm) long DNA molecules. On these molecules, the lateral bead fluctuations as well as the DNA length fluctuations were greatly reduced providing a better resolution of the 1/f-like noise component. The characteristic frequency of the DNA length fluctuations was shifted to much higher frequencies such that it could not be observed. To allow long time recordings of the position trajectories, the position trajectories were acquired at 1 kHz frame rate. When inspecting the position trajectories smoothed with a sliding box average to 1 Hz as well as the PSDs, we observed clear signatures of 1/f-like noise not only along the axial direction (z-direction) but in an (anti-)correlated manner also laterally along the magnetic field direction (y-direction, Fig. S7A). It was not observed in the transverse direction to the field (x-direction). When turning the magnetic field by 90°, the 1/f-like noise appeared again along the field (now x-direction) but not perpendicular to it (now y-direction). When turning the magnetic field to a total of 180°, the low frequency noise was again observed along the y-direction (not shown). When comparing the y- and z-trajectories for 0° magnet angle, the low frequency fluctuations appeared to be partially anti-correlated, while for 90° magnet angle the low frequency fluctuations appeared to be correlated (Fig. S7A).

Overall the observed behaviour can be understood by considering slow orientation fluctuations $\Delta\varphi(t)$ of the anisotropy axis in a plane perpendicular to the surface of the fluidic cell. These simultaneously change the axial and the lateral bead position along the field direction. The relative extend of these changes depends on the off-center attachment of the DNA. With φ being the angle at which the DNA is attached with respect to a horizontal plane through the bead center (Fig. 1C, main text), the changes of the lateral and the axial position due to an angular displacement $\Delta\varphi$ of the bead axis are given by:

$$\Delta y = R\Delta\varphi \sin\varphi$$

$$\Delta z = R\Delta\varphi \cos\varphi$$

with $\cos\varphi = R_{\perp}/R$. For $\varphi \approx 0°$, i.e. strong off-center attachment, the 1/f-like fluctuations would predominantly be seen along the axial direction, while for $\varphi \approx 90°$, i.e. small off-center attachment, they would predominantly be seen along the lateral direction. In agreement with this, we observed a similar magnitude of the 1/f-like fluctuations along z and y for an off-center attachment of 250 nm (Figs. S7A,B). For a bead with an off-center attachment of only 50 nm, the amplitude of the 1/f-like noise along z was about 15% of the amplitude along y (Figs. S7D,E). For $\varphi < 90°$ (bead center left of DNA attachment point), both $\sin\varphi$ and $\cos\varphi$ are positive, such that the position changes are correlated. For $\varphi > 90°$ (bead right of DNA attachment point), both position changes become anti-correlated.

Monitoring the 1/f-like position fluctuations along the field direction, provides the unique possibility to correct for their contribution along the axial direction. Applying such a correction by subtracting (correlated fluctuations) or adding (anti-correlated fluctuations) the scaled fluctuations along the field, provided a marked reduction of some of the axial 1/f-like noise particularly for the larger off-center attachment (Fig. S7C,F). Some of the fluctuations remain however. We attribute these to fluctuations of the anisotropy axis in a plane parallel to the surface. Thus, the correction applies only to one of the two possible orientations of axis fluctuations

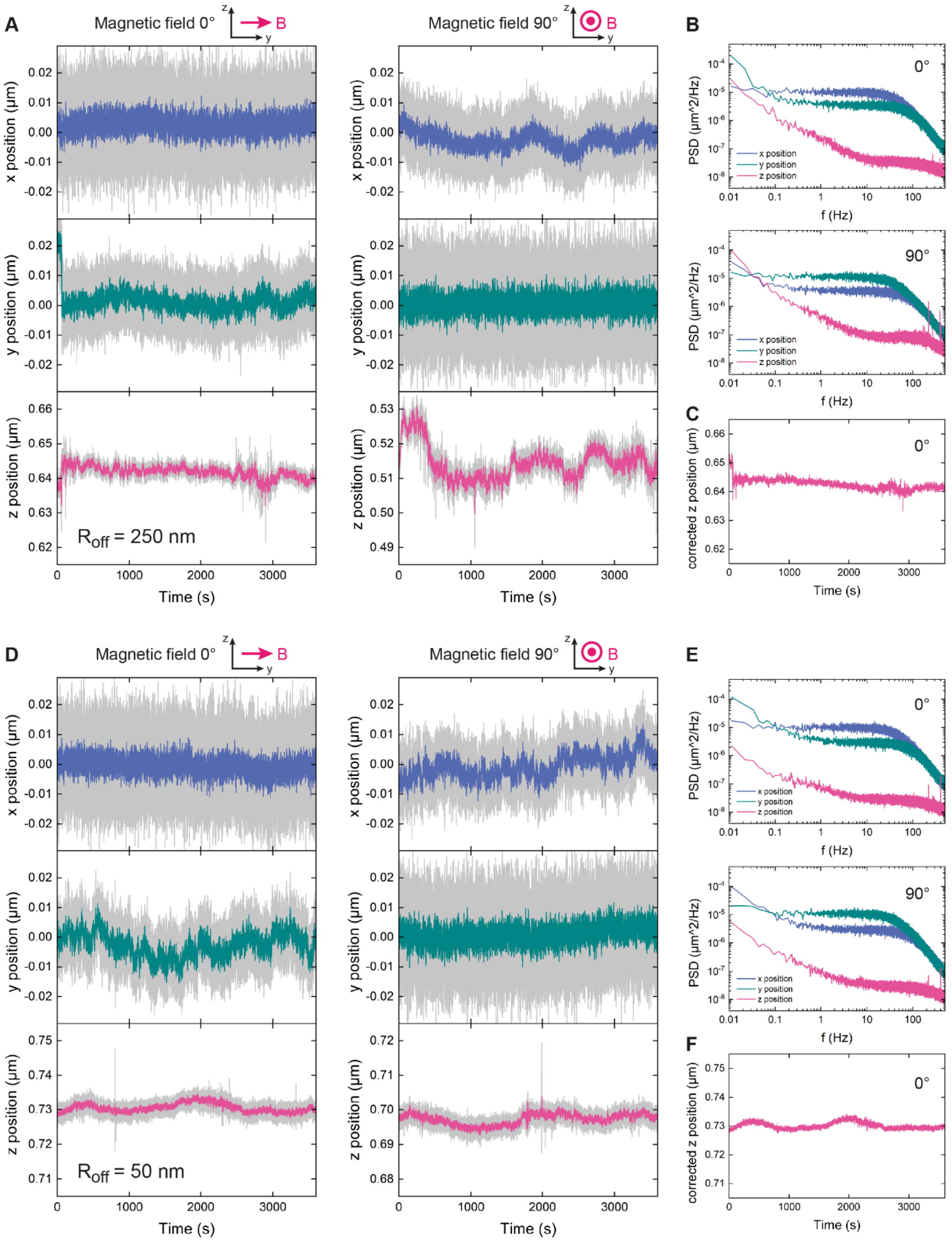

Figure S7: Low-frequency position noise probed along the axial and the lateral directions for a magnetic bead with $R_{\perp} = 250\ nm$ (A,B,C) and with $R_{\perp} = 50\ nm$ (D,E,F). Long trajectories of the of the magnetic bead position along x,y and z for the magnetic field oriented along y (left) and along x (right). Trajectories were recorded at 1kHz using 1-µm beads and 2.1 kbp long DNA molecules and a force of 6 pN. Gray lines show the data after sliding-box averaging to 10 Hz and coloured lines after filtering to 1 Hz. 1/f-like noise can be seen along the axial and laterally along the field direction but not in the transverse direction to the field. Axial and

lateral 1/f-like noise appear anti-correlated for the magnetic field along y and correlated for the magnetic field along x. B) PSDs of the position trajectories in A, revealing the strong 1/f-like noise along the axial direction and laterally along the field direction. C) Corrected z-position (magnetic field along y) obtained by adding the y-position data after sliding box averaging to 0.1 Hz and rescaling with a factor of 0.6 to the original data. Using this procedure part of the low-frequency fluctuations could be removed providing a flattened trajectory. D,E,F) Shown data is according to A,B,C. For the correction of the z position in F a scaling factor of 0.15 was applied.

Note S5 – Simulating the magnetization of magnetic beads

A) Magnetic moment, force and torque on a (super-)paramagnetic particle

When simulating the magnetization of superparamagnetic particles in magnetic tweezers experiments it is important to consider how magnetization, field and field gradient are connected to force and torque on the particle.

Isotropic superparamagnetic particle

We first consider an isotropic superparamagnetic particle with a constant magnetic moment m which is fully randomly oriented in absence of the magnetic field. In presence of a magnetic field $\vec{B}$, the potential energy of the particle with magnetic moment $\vec{m}$ is given as:

$$U = -\vec{m} \cdot \vec{B} = -mB\cos\theta \tag{S29}$$

where θ is the angle between the field and magnetic moment vectors. With this we can obtain the partition function by integration over all possible magnetic moment orientations:

$$Z = \int_0^{\pi}\int_0^{2\pi} e^{-U(\theta)/k_BT}\sin\theta\, d\varphi d\theta = \int_0^{\pi}\int_0^{2\pi} e^{mB\cos\theta/k_BT}\sin\theta\, d\varphi d\theta \tag{S30}$$

Solving the integral provides:

$$Z = 2\pi\frac{k_BT}{mB}\left(e^{mB/k_BT} - e^{-mB/k_BT}\right) = 4\pi\frac{k_BT}{mB}\sinh\left(\frac{mB}{k_BT}\right) \tag{S31}$$

The mean potential energy of the particle in the field can be calculated by

$$\langle U\rangle = -\frac{1}{Z}\frac{\partial}{\partial\beta}Z = -\frac{\partial}{\partial\beta}\ln Z \tag{S32}$$

with $\beta = 1/k_BT$, since potential energy U and β enter as a product in the exponent of the Boltzmann terms in Eq. S30. Inserting Z provides:

$$\langle U\rangle = -mB\left[\coth\left(\frac{mB}{k_BT}\right) - \frac{k_BT}{mB}\right] = -\underbrace{m\,L\left(\frac{mB}{k_BT}\right)}_{\langle m(B)\rangle}B \tag{S33}$$

where $L(x)$ is the Langevin function. The mean magnetic moment (along the field direction) is similarly given as:

$$\langle m(B)\rangle = \langle m\cos\theta\rangle = k_BT\frac{\partial}{\partial B}\ln Z \tag{S34}$$

since $m\cos\theta$ and B enter as a product in the exponents of the Boltzmann terms. Inserting provides the Langevin function for the magnetization of a super paramagnetic particle (see Eqn. S14 in Methods):

$$\langle m(B)\rangle = m\left(\coth\left(\frac{mB}{k_BT}\right) - \frac{k_BT}{mB}\right) = m\,L\left(\frac{mB}{k_BT}\right) \tag{S35}$$

The mean potential energy of the isotropic superparamagnetic particle is thus given by the intuitive expression:

$$\langle U\rangle = -\langle m(B)\rangle B \tag{S36}$$

The free energy of the particle inside the field is given as:

$$G = -k_BT\left[\ln Z(B) - \ln\underbrace{Z(B=0)}_{4\pi}\right] \tag{S37}$$

Inserting Z provides:

$$G = -k_BT\ln[Z(B)/4\pi] = -k_BT\ln\left[\frac{k_BT}{mB}\sinh\left(\frac{mB}{k_BT}\right)\right] \tag{S38}$$

Thus, the free energy becomes with increasing field B larger than the mean potential energy, since the initial random orientation of the magnetic moment becomes increasingly confined along the field direction (Fig. S8).

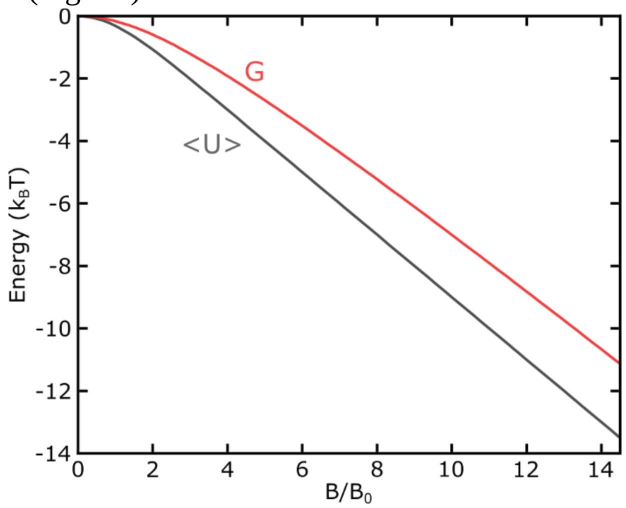

Figure S8: Mean potential energy and free energy of an isotropic superparamagnetic particle inside a magnetic field. The field is given in units of $B_0 = k_B T/m_{tot}$.

In case of a field gradient, the field and thus the free energy becomes position-dependent i.e. $B = B(\vec{r})$. This causes a force on the particle, which is given by the negative gradient of the free energy:

$$\vec{F} = -\vec{\nabla}G = k_B T \frac{\vec{\nabla}Z(B)}{Z(B)} = \underbrace{\frac{k_B T}{Z(B)} \frac{\partial Z}{\partial B}}_{\langle m(B) \rangle} \vec{\nabla}B = \underbrace{k_B T \frac{\partial}{\partial B} \ln Z(B)}_{\langle m(B) \rangle} \vec{\nabla}B \tag{S39}$$

where the expressions on the right side were obtained by inserting Eq. S37 for the free energy. This expression contains the mean magnetic moment along the field (Eq. S34), such that the force of an isotropic magnetic particle in a field gradient is simply given by:

$$\vec{F} = \langle m(B) \rangle \vec{\nabla}B \tag{S40}$$

The torque on the particle would be given as:

$$\Gamma(B, \theta_{NP}) = -\frac{dG(B)}{d\theta_{NP}} = 0 \tag{S41}$$

It is zero, since for an isotropic nanoparticle the free energy of magnetization is independent of the particle orientation.

Anisotropic superparamagnetic nanoparticle (in two dimensions)

Mean magnetic moment, force and torque considering thermal fluctuations

Now we consider an anisotropic superparamagnetic particle. For simplicity we consider only magnetic moment orientations within the plane spanned by the magnetic field vector and the anisotropy axis (see Fig. 5 main text). For an anisotropic nanoparticle whose anisotropy axis forms an angle θ_{NP} with respect to the applied magnetic field, the potential energy is given as (see main text):

$$U = C \sin^2(\theta_{NP} - \theta_2) - mB \cos\theta_2 \tag{S42}$$

with $C = KV/2$ (where K is the anisotropy constant and V the volume of the nanoparticle) and θ_2 being the angle formed between the magnetic moment and the magnetic field (see Fig. 5

main text). For in-plane rotations of the magnetic moment vector, the partition function is given as:

$$Z = \int_0^{2\pi} e^{[mB\cos\theta_2 - C\sin^2(\theta_{NP}-\theta_2)]/k_BT}\, d\theta_2 \tag{S43}$$

To get the mean magnetic moment along the field direction, we can write:

$$\langle m_{par}(B,\theta_{NP})\rangle = \frac{1}{Z}\int_0^{2\pi} m\cos\theta_2\, e^{[mB\cos\theta_2 - C\sin^2(\theta_{NP}-\theta_2)]/k_BT}\, d\theta_2 \tag{S44}$$

which can be rewritten to:

$$\langle m_{par}(B,\theta_{NP})\rangle = \frac{1}{Z}\int_0^{2\pi} k_BT\frac{\partial}{\partial B} e^{[mB\cos\theta_2 - C\sin^2(\theta_{NP}-\theta_2)]/k_BT}\, d\theta_2 \tag{S45}$$

By moving the differentiation out of the integral, this can be transformed to:

$$\langle m_{par}(B,\theta_{NP})\rangle = k_BT\frac{1}{Z}\frac{\partial}{\partial B}Z = k_BT\frac{\partial}{\partial B}\ln Z = -\frac{\partial}{\partial B}G(B,\theta_{NP}) \tag{S46}$$

where $G(B,\theta_{NP})$ is the free energy of the nanoparticle in the field. The force on the nanoparticle in a field gradient is given by:

$$\vec{F}(\theta_{NP}) = -\vec{\nabla}G(B,\theta_{NP}) = -\frac{\partial}{\partial B}G(B,\theta_{NP})\,\vec{\nabla}B = \langle m_{par}(B,\theta_{NP})\rangle\,\vec{\nabla}B \tag{S47}$$

i.e. it is again given by the mean magnetic moment along the field multiplied with the field gradient. If the particle is free to rotate or consists of a large ensemble of superparamagnetic nanoparticles with random orientations, one averages over the force as well as the mean magnetic moment over θ_{NP}. For symmetry reasons perpendicular magnetic moment components average out, such that one can write:

$$\vec{F} = \langle m(B)\rangle\,\vec{\nabla}B \tag{S48}$$

The torque on a particle with a given orientation θ_{NP} is given as:

$$\Gamma(B,\theta_{NP}) = -\frac{\partial G(B,\theta_{NP})}{\partial\theta_{NP}} \tag{S49}$$

It is non-zero, if the particle anisotropy axis is not parallel to the field.

$$\langle m_{perp}(B,\theta_{NP})\rangle = \frac{1}{Z}\int_0^{2\pi} m\sin\theta_2\, e^{[mB\cos\theta_2 - C\sin^2(\theta_{NP}-\theta_2)]/k_BT}\, d\theta_2 \tag{S50}$$

with $\theta_1 = \theta_2 - \theta_{NP}$ we get:

$$\langle m_{perp}(B,\theta_{NP})\rangle = \frac{1}{Z}\int_{-\theta_{NP}}^{2\pi-\theta_{NP}} m\sin\theta_1\, e^{[mB\cos(\theta_1+\theta_{NP}) - C\sin^2(-\theta_1)]/k_BT}\, d\theta_1 \tag{S51}$$

which can be transformed to

$$\langle m_{perp}(B,\theta_{NP})\rangle = -\frac{1}{Z}\int_0^{2\pi}\frac{k_BT}{B}\frac{\partial}{\partial\theta_{NP}} e^{[mB\cos(\theta_1+\theta_{NP}) - C\sin^2(-\theta_1)]/k_BT} d\theta_1 \tag{S52}$$

since both trigonometric functions are periodic with respect to the integration interval. By moving the differentiation out of the integral and back-substituting θ_2, this can be transformed to:

$$\langle m_{senk}(B,\theta_{NP})\rangle = -\frac{k_BT}{B}\frac{1}{Z}\frac{\partial Z}{\partial\theta_{NP}} = -\frac{k_BT}{B}\frac{\partial}{\partial\theta_{NP}}\ln Z = \frac{1}{B}\frac{\partial G(B,\theta_{NP})}{\partial\theta_{NP}} \tag{S53}$$

Using the torque expression from above we get thus the simple relation:

$$\Gamma(B,\theta_{NP}) = -\langle m_{senk}(B,\theta_{NP})\rangle\, B \qquad \text{(S54)}$$

i.e. as for a fixed dipole orientation, the torque is given by the vector product of mean magnetic moment and B-field:

$$\vec{\Gamma}(B,\theta_{NP}) = \langle \vec{m}(B,\theta_{NP})\rangle \times \vec{B} \qquad \text{(S55)}$$

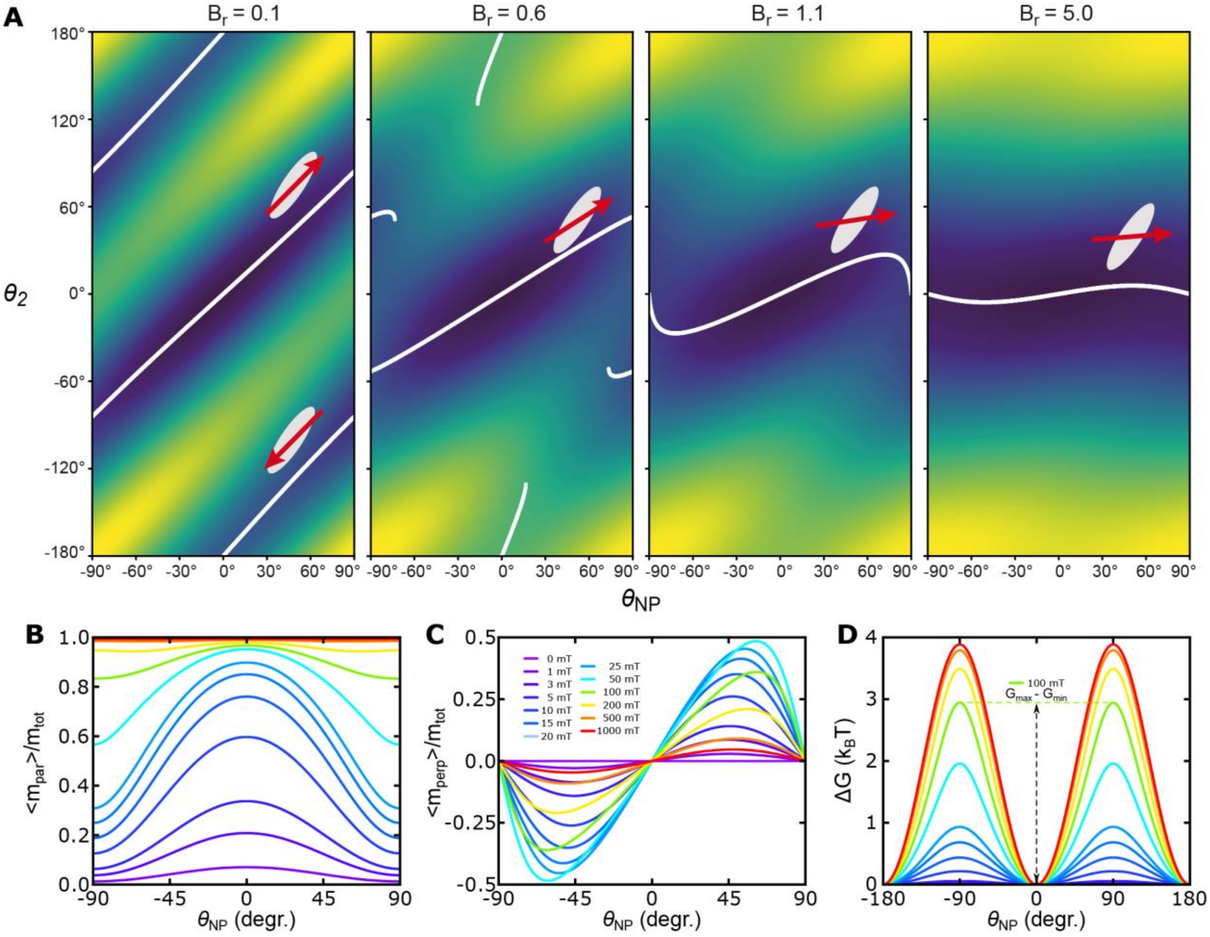

Figure S9: Potential energy, components of the average magnetic moment and free energy of a nanoparticle as function of its orientation θ_{NP} to the magnetic field B. (A) Reduced potential energy as function of the nanoparticle orientation θ_{NP} and the magnetic moment orientation θ_2 plotted for different reduced fields $B_r = B/(K/M)$ for which the Stoner-Wohlfarth model simplifies to $U/(VK) = 0.5\sin^2(\theta_{NP} - \theta_2) - B_r \cos\theta_2$. White lines mark the positions of local minima of the potential energy with respect to θ_2. For $B_r < 0.5$ two minima, representing two almost opposing magnetic moment orientations along the NP axis, are obtained for all NP orientations. For $0.5 < B_r < 1.0$ a second minimum is only obtained for some NP orientations, while for $B_r > 1.0$ only a single minimum is found. At such high fields the magnetic moment aligns strong with the field than with the NP axis. Cartoons illustrate the typical magnetic moment orientations. For $M = 314$ kA/m and $K = 30$ kJ/m^3, $B_r = 1$ corresponds to a magnetic field of $B = 96\, mT$. (B,C,D) Components of the average magnetic moment parallel to the magnetic field $\langle m_{par}\rangle$ and perpendicular to the magnetic field $\langle m_{perp}\rangle$ as well as the free energy of the magnetic dipole in the field (from left to right). The quantities were plotted for different magnitc fields (see legend in C). The barrier height of the energy landscape in D was determined as $G_{max} - G_{min}$ (indicated for the 100 mT curve with a dashed line). The shown calculations used an anisotropy constant of 30 kJ/m³ and an NP radius of 6.36 nm.

Torque and torsional stiffness neglecting thermal fluctuations

To derive simple estimates of the torque and the torsional stiffness of a NP in the magnetic field, we now assume the anisotropy to be sufficiently strong to allow only small displacements of the magnetic moment from its axis. Furthermore, the particle shall be free to rotate and the field shall be sufficiently strong such that the NP and its magnetic moment align strongly with the magnetic field. In this case one can apply the small angle approximation $\theta_{NP}, \theta_2 \ll 1$ [15, 16]. For a given θ_{NP} one can thus obtain the location of the minimum of the potential energy function (Eq. S42) from:

$$0 = \frac{\partial U}{\partial \theta_2} = -2C \sin(\theta_{NP} - \theta_2) \cos(\theta_{NP} - \theta_2) + mB \sin \theta_2 \approx -2C(\theta_{NP} - \theta_2) + mB\, \theta_2 \qquad \text{(S56)}$$

This provides the most probably θ_2 for the orientation of the magnetic moment:

$$\theta_2 = \frac{2C}{2C + mB} \theta_{NP} \qquad \text{(S57)}$$

The torque on the NP in the small angle approximation is given as:

$$\Gamma(B, \theta_{NP}) = -\frac{\partial U}{\partial \theta_{NP}} \approx -2C(\theta_{NP} - \theta_2) = -\frac{2CmB}{2C + mB} \theta_{NP} \qquad \text{(S58)}$$

The torsional stiffness is then given as:

$$k_{tor} \approx \frac{2CmB}{2C + mB} = \frac{KV\, B}{K/M + B} \qquad \text{(S59)}$$

applying $C = KV/2$ and $m = MV$. To account to some extent for magnetic moment fluctuations at low magnetic fields, Oene et al.[16] used a field-dependent magnetic moment $\langle m(B) \rangle$ described either by a Langevin function or a hyperbolic tangent, the latter describing the magnetization of a 2-state paramagnet. Applying the Langevin function gave good agreement for low anisotropy constants while the hyperbolic tangent gave good agreement for high anisotropy constants. Overall one gets at low magnetic fields an increasing torsional stiffness $k_{tor} \approx \langle m(B) \rangle B$. At high fields the torsional stiffness plateaus at $k_{tor} = KV$.

B) Parameters used in the simulations of magnetic beads

i) Magnetization (per volume) of maghemite nanoparticles

The magnetization (magnetic moment per volume) of the maghemite nanoparticles was obtained from previous measurements of the magnetization of 1-µm MyOne and 2.8-µm M280 beads [4]. It was calculated by firstly converting the iron content of the beads to the maghemite content. The magnetization per sample mass $M_{m,bead}$ divided by the maghemite content then provides the magnetization per maghemite mass $M_{m,Fe_2O_3} = M_{m,bead}/(m_{Fe_2O_3}/m_{bead})$. Multiplication with the maghemite density of $\rho_{Fe_2O_3} = 4.9\ g/cm^3$ yields then the volume magnetization of maghemite $M_{V,Fe_2O_3} = M_{m,Fe_2O_3}\rho_{Fe_2O_3} = 314\ kA/m$. This value was obtained for MyOne as well as M280 beads. The total volume of the magnetic material in different bead types was also derived from that data and was $V_{MyOne} = 7.68 \cdot 10^{-20}\ m^3$ and $V_{M280} = 5.73 \cdot 10^{-19}\ m^3$ for 1-µm and 2.8-µm beads respectively. All relevant values of these calculations are listed in Table S2. We note that previous modelling approaches of the nanoparticle magnetization used $M_{V,Fe_2O_3} = 43.3\ kA/m$ for MyOne and $M_{V,Fe_2O_3} = 27.9\ kA/m$ for M270 beads[16] as volume

magnetization. These values were however derived from magnetization measurements of whole beads and severely underestimate the volume magnetization of single nanoparticles. This may have introduced considerable errors, by overestimating the anisotropy constant in the potential energy function.

Table S2: Magnetic and size parameters of the different beads.

Bead	m_{Fe}/m_{bead} (mg/g)	$M_{m,bead}$ (Am²/kg)		r_{bead} (µm)	ρ_{bead} (g/cm³)	V_{tot} (m³)	r_{NP} (nm)	N
MyOne	255	23.5		0.5	1.7	$7.68\cdot10^{-20}$	3.92	$3.05\cdot10^{5}$
M280	118	10.8		1.4	1.4	$5.73\cdot10^{-19}$	3.92	$2.28\cdot10^{6}$
	$m_{Fe_2O_3}/m_{bead}$ (mg/g)	M_{m,Fe_2O_3} (Am²/kg)	M_{V,Fe_2O_3} (kA/m)			V_{tot} (m³)	r_{NP} (nm)	N
MyOne	365	64.4	315			$7.68\cdot10^{-20}$	6.36	$7.10\cdot10^{4}$
M280	169	63.9	313			$5.73\cdot10^{-19}$	5.84	$6.85\cdot10^{5}$

Fe denotes the iron content and Fe_2O_3 the maghemite content per bead mass. $M_{m,bead}$ is the magnetization per bead mass, M_{m,Fe_2O_3} is the magnetization per maghemite mass, M_{V,Fe_2O_3} is the magnetization per maghemite volume. r_{bead} and ρ_{bead} are the radii and density of the beads. V_{tot} is the total volume of magnetic material in a bead, r_{NP} is radius of a spherical NP and N is the number of NPs per bead. Values shaded in grey were previously publishedf[17], blue shaded values are derived values as used in our simulations.

ii) Nanoparticle radii

Fonnum et al.[17] found that the radii of maghemite nano-particles in 2.8-µm M280 beads was about 4 nm using SEM images (see Table S2). The nanoparticle size has a considerable influence on the magnetization curves, as can be easily seen from the characteristic field $B_0 = k_B T/m = k_B T/MV$ that is part of the Langevin function (19). The characteristic fields of MyOne and M280 magnetic beads have previously been determined as 12 mT and 15.5 mT respectively by fitting Langevin functions to experimental magnetization curves[18, 19]. Calculating the NP radii from the experimental values for B_0 using the underestimated volume magnetization in previous modelling (see point i) would yield severely overestimated nanoparticle radii. Instead using the obtained volume magnetization of maghemite M_{m,Fe_2O_3} (Table S2) yielded radii of $r_{NP,MyOne} = 6.36\ nm$ and $r_{NP,M280} = 5.84\ nm$ in much better agreement with the SEM data. Therefore, we used these radii in our simulations and also applied them to calculate the number of NPs per bead to maintain the correct volume of magnetic material (Table S2).

iii) Parametrization of the anisotropy constant

The anisotropy of the NPs determines the torsional stiffness of the simulated bead in the field. We applied NP anisotropy constants of 4.7, 13 and 30 kJ/m³ in our simulations to achieve a good match with the experimentally data. K =4.7 kJ/m³ represents the experimental magnetocrystalline anisotropy constant of crystalline maghemite[20].

To estimate the contribution of the shape anisotropy, we calculated the anisotropy arising from dipole-dipole interactions of two adjacent isotropic NPs. The potential energy of two interacting magnetic moments is approximately given by:

$$U = -\frac{\mu_0 \mu_r (MV)^2}{4\pi r_{dip}^3}\left(2\cos(\theta_{1,1})\cos(\theta_{1,2}) - \sin(\theta_{1,1})\sin(\theta_{1,2})\cos(\varphi)\right) - MVB(\cos\theta_{2,1} + \cos\theta_{2,2}) \quad \text{(S60)}$$

Where $r_{dip} = 2r_{NP}$ is the distance between the centers of the adjacent NPs, $\theta_{1,1}$ and $\theta_{1,2}$ the angles between the line that connects the center of the two particles and the magnetic moment of the respective NP and φ the angular difference of the azimuthal angles of the magnetic moments around this line. $\theta_{2,1}$ and $\theta_{2,2}$ are the angles between the magnetic field and the magnetic moment of the respective NP. For simplification, we assume that both magnetic point mainly in the same direction and that the deviation of the magnetic moments occurs in the plane formed by dimer axis and magnetic field. In analogy to Fig. 5A main text, we thus have $\theta_{2,1} = \theta_{2,2} = \theta_2$ and $\theta_{1,1} = \theta_{1,2} = \theta_{dimer} - \theta_2$. Using that the nanoparticle volume is given by $V = (4/3)\,\pi r_{NP}^3$, $\mu_r \approx 1$ and further simplification provides for the potential energy of 2 adjacent isotropic NPs in the magnetic field:

$$U \approx \frac{\mu_0 \mu_r M^2 V}{8}\sin^2(\theta_{dimer} - \theta_2) - 2MVB\cos\theta_2 \quad \text{(S61)}$$

This expression is of the same form as the potential energy of a single anisotropic NP, such that one can estimate an apparent anisotropy (per particle volume) for magnetic dipole-dipole interactions of:

$$K_{app,dimer} = \frac{1}{8}\mu_0 \mu_r M^2 \quad \text{(S62)}$$

with this we obtain an apparent anisotropy of K =13 kJ/m³. To account for larger NP clusters that can exist[17] we also used an increased anisotropy of K =30 kJ/m³.

In Fig. S10 we present a comparison of the torsional stiffness of a single NP for the different anisotropy constants used in our model (see main text), the result of a numerical calculation of the dipole-dipole model allowing independent dipole moment fluctuations of the 2 NPs in 2D and a previous modelling done by Oene et al.[16]. Considering a NP with an adopted anisotropy constant of K =13 kJ/m³ provides a good approximation for the torsional stiffness of two adjacent isotropic NPs.

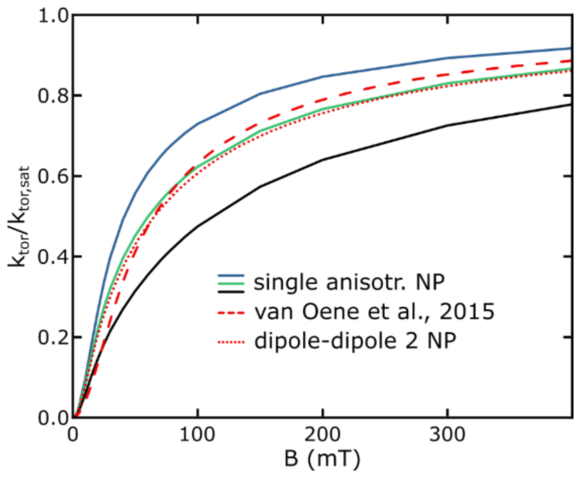

Figure S10: Torsional stiffnesses of a single NP as function of the magnetic field (normalized by its saturation value). Shown are equilibrium calculations for single particles with a radius

of 5.84 nm and anisotropy constants of 4.7 kJ/m³ (blue line), 13 kJ/m³ (green line) and 30 kJ/m³ (black line) as well as equilibrium calculations for two adjacent isotropic NPs that interact by dipole-dipole interactions with independent dipole angles (red dotted line, see Note S5B iii). For comparison, the heuristic composite model from van Oene et al.[16] with an anisotropy constant of 13 kJ/m³ that does not include fluctuations is shown (red dashed line). Notably, there is a good agreement between the torsional stiffnesses calculated for the single NP with K =13 kJ/m³ and the NP dimer with dipole-dipole interactions over the whole field range. The model by van Oene et al. shows significant deviations at low fields but good agreement at high fields.

C) Limitations from modelling magnetic moment fluctuations in 2D

To limit numerical efforts when superimposing the free energy landscapes of a large number of NPs in the bead simulations, we restricted the orientation of the magnetic moments to a plane spanned by the anisotropy axis and the magnetic field vectors. This removes one degree of freedom, which will affect the magnetization curves of the beads as well as the obtained torsional stiffnesses. In the following, the influence of this limitation will be discussed.

For fluctuations in 3D and in absence of anisotropy, the mean magnetic moment of a NP or a magnetic bead along the field is given by a Langevin function that is of the form (see Note S5, Eq. S35):

$$\langle m(B)\rangle = m_{tot}\left(\coth\left(\frac{B}{B_0}\right) - \frac{B_0}{B}\right) \qquad \text{(S63)}$$

where $m_{tot} = N\,m$ is the saturation magnetic moment of N NPs, m the magnetic moment of a single NP and $B_0 = k_B T/m$ is the characteristic field. In analogy to an entropic spring[21], the linear part of the magnetization curve at low fields ($B \ll B_0$) is given by:

$$\langle m(B)\rangle \approx \frac{m_{tot} m}{f\,k_B T} B = \frac{m_{tot}}{f}\frac{B}{B_0}$$

where f denotes the degree of freedom of the fluctuations, with $f = 3$ in 3D and $f = 2$ in 2D. Thus, when limiting the fluctuations to 2D the slope of the magnetization curve is 3/2 steeper than for the 3D case. It is thus easier to magnetize a particle in 2D compared to 3D.

Fig. S13 compares the experimental magnetization curves of 1 µm and 2.8 µm beads (shown together with the corresponding Langevin fit) with our simulations for these bead types. For the same B_0 as obtained from the Langevin fits, our simulations exhibited a steeper initial slope than the measurements in agreement with the limitation of magnetic moment fluctuations in 2D. When numerically calculating the magnetization curves in absence of anisotropy for 2D fluctuations, we obtained good agreement with the whole bead simulations. The small deviations can be attributed to the influence of the anisotropies of the nanoparticles. Depending on the NP orientation, the initial slope of the magnetization curves can for anisotropic NPs be larger (e.g. $\theta_2 = 0°$) or smaller (e.g. $\theta_2 = 90°$) than for isotropic NPs. The good agreement of the bead simulations with the isotropic NP simulations in 2D suggests that the orientation effects cancel each other quite effectively. This can explain why the measured magnetization of 1-µm beads follows a Langevin behaviour (Figure S13A). In contrast, For 2.8 µm beads larger deviations are observed, suggesting an increased influence of anisotropies, e.g. due to larger NP aggregates with increased dipole-dipole interactions.

We expect that the limitation of our simulations to 2D will also provide deviations for the torsional stiffness when compared to simulations in 3D. We expect that the torsional stiffness is mainly determined by NPS with orientations that do not deviate too much from the plane

spanned by field vector and bead anisotropy axis. NPs that approach perpendicular orientations will barely contribute to the torsional stiffness. Overall, in the 3D situation a smaller number of NPs will contribute to the torsional stiffness, such that it will be lower than modelled in this work. We expect a reduction of our values by a small factor, which will however not influence the order of magnitude of our simulations results.

D) Results of bead simulations

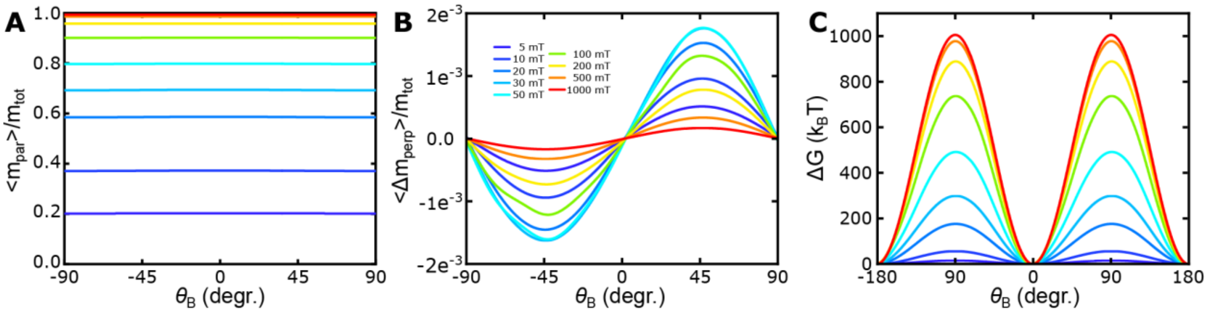

Figure S11: Simulation of the magnetization of a 1-µm magnetic bead as function of the applied field (see legend in B). (A) Component of the average magnetic moment $\langle m_{par} \rangle$ parallel to the magnetic field. The bead has a very small anisotropy in contrast to a single NP, such that the orientation-dependence of the parallel magnetization component is very small. (B) Component of the average magnetic moment $\langle m_{perp} \rangle$ perpendicular to the magnetic field. (C) Free energy of the bead magnetization as function of the bead angle θ_B with respect to the field. The shown calculations used an anisotropy constant of 30 kJ/m³ and an NP radius of 6.36 nm.

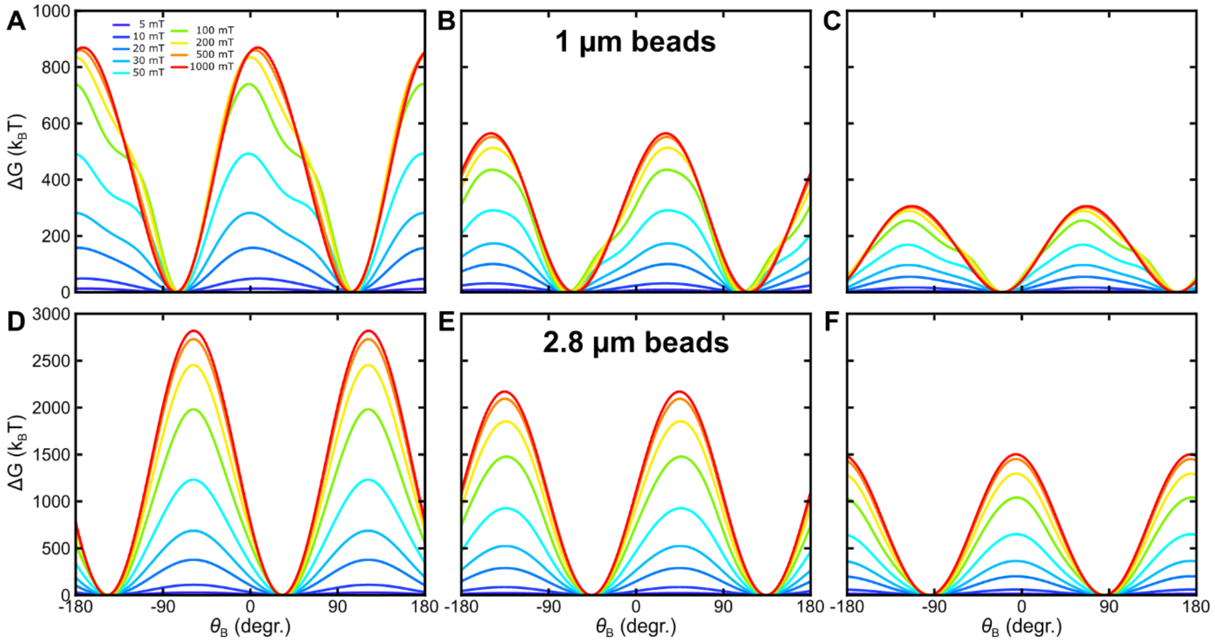

Figure S12: Simulated free energies of 1-µm (A,B,C) and 2.8-µm (D,E,F) magnetic beads as function of the bead orientation θ_B. Different colours show the free energy profiles at different magnetic fields (see legend in A). Each plot (A to F) shows data of a single bead, i.e. for a particular set of randomly orientated NPs. For each curve the minimum free energy was set to zero to reveal its modulation by the applied field. The pronounced minima of the free energy function every 180° reveals for all simulated beads the formation of a single well-defined anisotropy axis. The simulations used an anisotropy constant of 30 kJ/m³. It is evident that the shape of the energy function as well as the barrier heights given by $G_{max} - G_{min}$ depend on the particular distribution of the NPs.

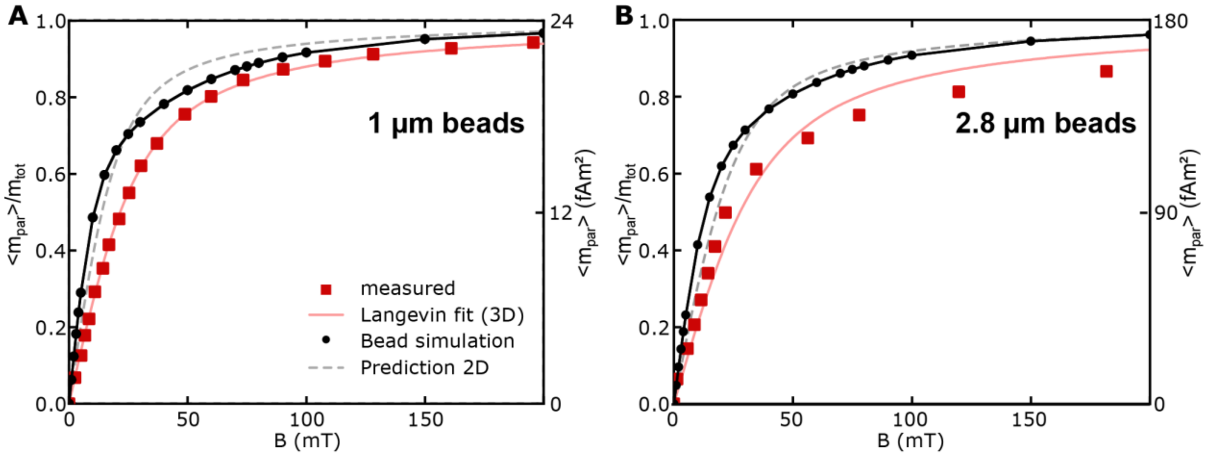

Figure S13: Mean magnetic moment parallel to the field as function of the applied field for (A) 1-µm and (B) 2.8-µm magnetic beads. Shown are the results of the simulations (black circles and lines) and measured magnetization curves (red squares) for 1-µm and 2.8-µm beads[18, 19]. The light red line is a Langevin-fit according to Eq. S35 (Note S5, assuming 3D fluctuations) to the measured data yielding $B_0^{1\,\mu m} = 12\,mT$ and $B_0^{2.8\,\mu m} = 15.5\,mT$. The grey dashed line shows a numerically calculated magnetization curve for isotropic NPs assuming that the magnetic moment fluctuations are limited to 2D (see Note S5C). The magnetic moments were normalized by their saturation values at high magnetic fields (left axes). Additionally, the absolute magnetic moment per bead was given for the simulations and the measurements (right axes).

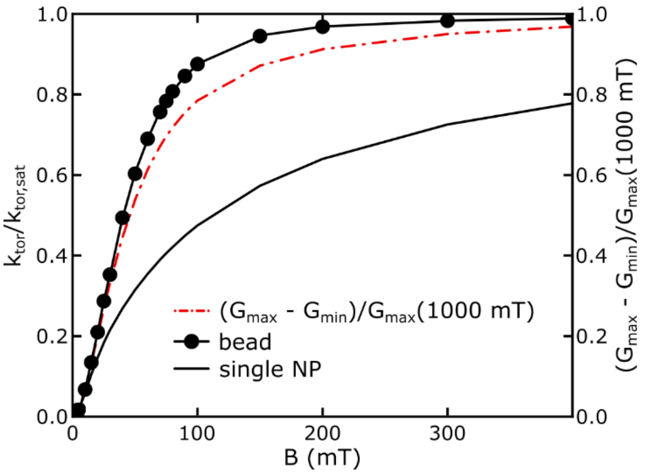

Figure S14: Simulated torsional stiffnesses of a 2.8-µm bead as function of the magnetic field (black line and circles, normalized by its saturation value). Shown is the average over several simulations, with an anisotropy constant of 30 kJ/m³. The black solid line shows the torsional stiffness of a single NP with the same anisotropy constant. The red dashed dotted line represents the maximum free energy difference $G_{max} - G_{min}$ of the free energy landscape of a NP (see Fig. S9) as function of the field normalized by its saturation value at high magnetic fields. The field-dependence of the torsional stiffness of the bead is much better described by the maximum free energy difference than the torsional stiffness of a single MP.

E) Results of MRX measurements

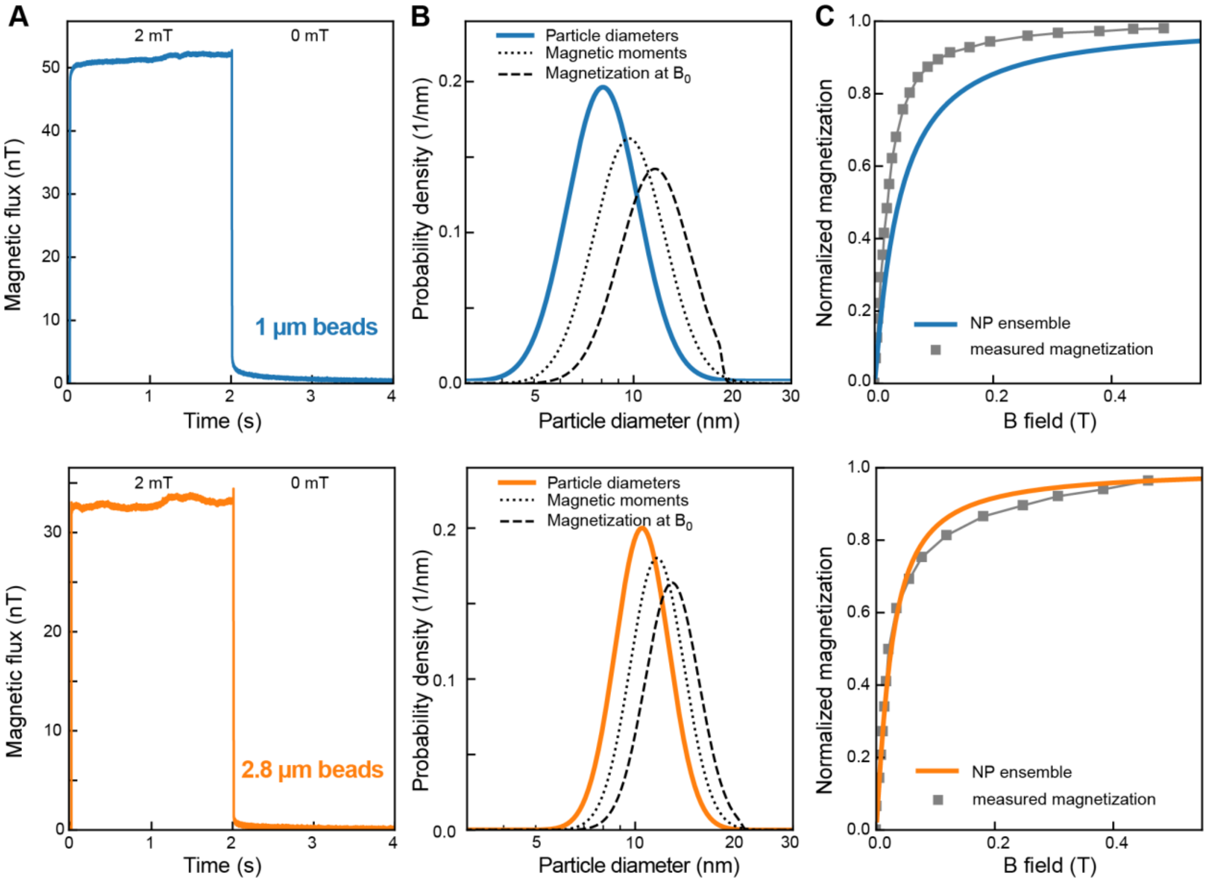

Figure S15: Flux gate MRX measurements and analysis results. (A) Full magnetic flux trajectories of the magnetized bead samples obtained by suddenly applying a field of B_0 = 2 mT at 0 s and a field of 0 mT at 2 s. Fig. 7A, main text, shows a zoom onto the relaxation part of the trajectories from 2 to 4 s. (B) Log-normal distributions of the NP diameters $p_d(d)$ calculated for the fit parameters from Fig. 7A (solid lines). The dotted lines show the relative contribution of the NPs to the total magnetic moment of the bead as function of diameter. It is derived by normalizing $p_m(d) = MVp_d(d)$, where M is the volume magnetization and $V = (\pi/6)d^3$ the volume of the NP. Compared to the size distribution it is shifted to the right, since the magnetic moment of an NP increases with d^3. The dashed lines show the relative contribution of the NPs to the magnetization of the bead at B_0 = 2 mT as function of diameter. It is derived by normalizing $p_{mag}(d) = p_m(d)\, L(MV, B_0)\, p_{ini}(d, B_0)$, where L is the Langevin function and $p_{ini}(d, B_0, t_{mag})$ is the fraction of NPs that become oriented within the magnetization time $t_{mag} = 2s$ along the field. Overall the distribution is shifted to the right since at 2 mT small particles do not yet become magnetized as given by $L(MV, B_0)$. The right side of the distribution appears truncated since with increasing size of the nanoparticles the energy barriers from the anisotropy increase and prevent alignment of the nanoparticles with the field. (C) Predicted magnetization curves for the obtained log-normal distributions of NP diameters (colored solid lines) compared to experimental magnetization curves for both bead types[18, 19].

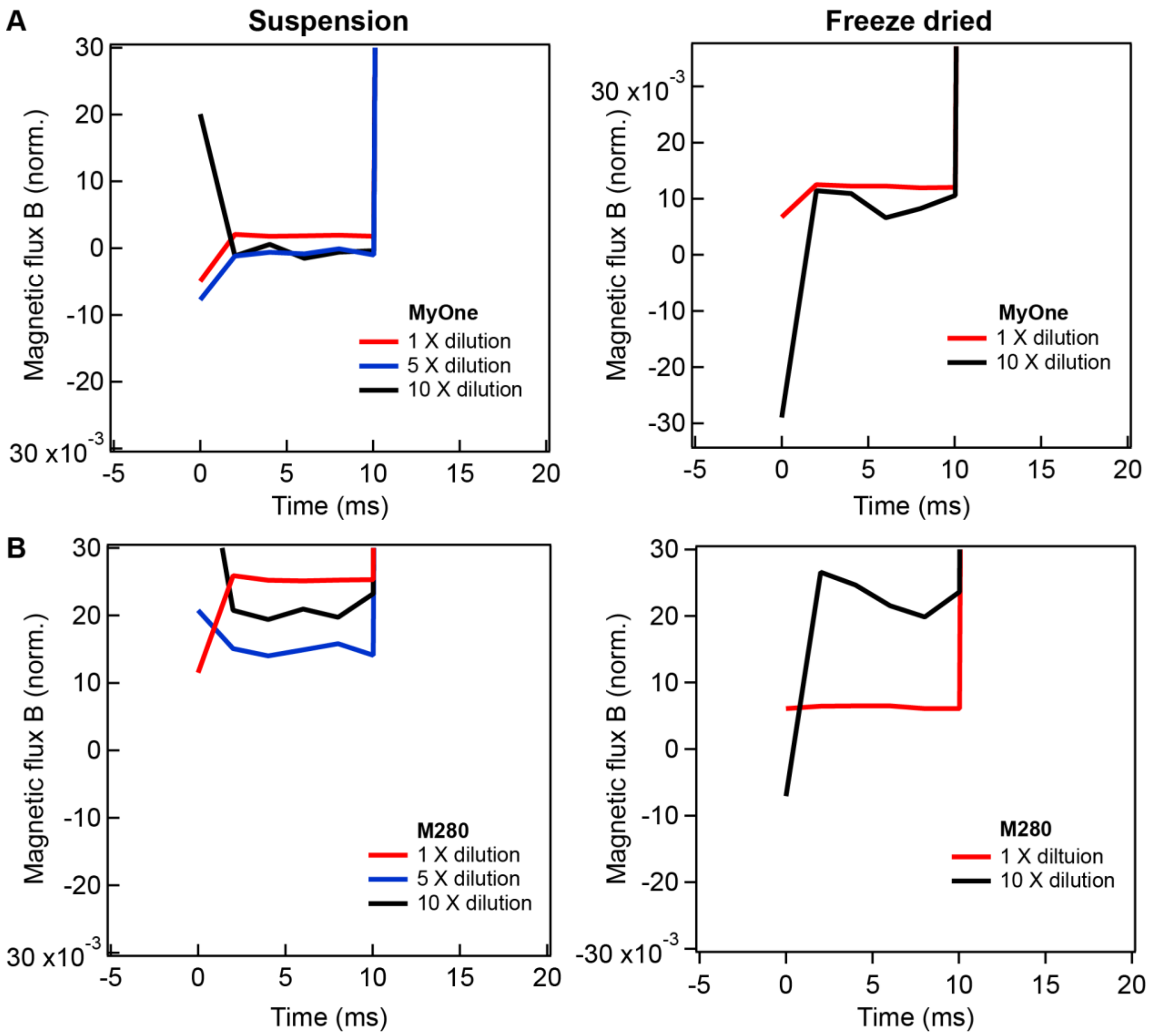

Figure S16: Enlarged view into MRX curves at zero field before the start of the magnetization at 10 ms for (A) 1-µm and (B) 2.8-µm magnetic beads measured for freely diffusing beads, where the particle magnetization can additionally relax via rotational diffusion (left) and freeze-dries bead, immobilized beads, which can only undergo Néel relaxation (right). Notably, freely diffusing 1 µm beads exhibit the weakest initial magnetization, compared to the immobilized samples and the larger M280 samples. This suggests that part of the magnetization of the beads decays on very long times scales (≫min). Only in case of 1 µm beads, the rotational diffusion allows a faster relaxation.

References

(1) Luzzietti, N.; Knappe, S.; Richter, I.; Seidel, R. Nicking enzyme-based internal labeling of DNA at multiple loci. *Nat. Protoc.* **2012**, *7*, 643–653.

(2) Rutkauskas, M.; Songailiene, I.; Irmisch, P.; Kemmerich, F. E.; Sinkunas, T.; Siksnys, V.; Seidel, R. A quantitative model for the dynamics of target recognition and off-target rejection by the CRISPR-Cas Cascade complex. *Nat. Commun.* **2022**, *13*, 7460.

(3) Klaue, D.; Seidel, R. Torsional stiffness of single superparamagnetic microspheres in an external magnetic field. *Phys. Rev. Lett.* **2009**, *102*, 028302.

(4) Huhle, A.; Klaue, D.; Brutzer, H.; Daldrop, P.; Joo, S.; Otto, O.; Keyser, U. F.; Seidel, R. Camera-based three-dimensional real-time particle tracking at kHz rates and Ångström accuracy. *Nat. Commun.* **2015**, *6*, 5885.

(5) Daldrop, P.; Brutzer, H.; Huhle, A.; Kauert, D. J.; Seidel, R. Extending the range for force calibration in magnetic tweezers. *Biophys. J.* **2015**, *108*, 2550–2561.

(6) Bouchiat, C.; Wang, M. D.; Allemand, J.; Strick, T.; Block, S. M.; Croquette, V. Estimating the persistence length of a worm-like chain molecule from force-extension measurements. *Biophys. J.* **1999**, *76*, 409–413.

(7) Wang, M. D.; Yin, H.; Landick, R.; Gelles, J.; Block, S. M. Stretching DNA with optical tweezers. *Biophys J.* **1997**, *72*, 1335–1346.

(8) Gittes, F.; Schmidt, C. F. *Methods in Cell Biol.*; Academic Press, 1998; Vol. 55; pp 129–156.

(9) Berg-Sørensen, K.; Flyvbjerg, H. Power spectrum analysis for optical tweezers. *Rev. Sci. Instrum.* **2004**, *75*, 594.

(10) Nørrelykke, S. F.; Flyvbjerg, H. Power spectrum analysis with least-squares fitting: amplitude bias and its elimination, with application to optical tweezers and atomic force microscope cantilevers. *Rev. Sci. Instrum.* **2010**, *81*, 075103.

(11) Ludwig, F.; Heim, E.; Schilling, M. Characterization of superparamagnetic nanoparticles by analyzing the magnetization and relaxation dynamics using fluxgate magnetometers. *J. Appl. Phys.* **2007**, *101*, 113909.

(12) Lansdorp, B. M.; Saleh, O. A. Power spectrum and Allan variance methods for calibrating single-molecule video-tracking instruments. *Rev. Sci. Instrum.* **2012**, *83*, 025115.

(13) te Velthuis, A. J. W.; Kerssemakers, J. W. J.; Lipfert, J.; Dekker, N. H. Quantitative guidelines for force calibration through spectral analysis of magnetic tweezers data. *Biophys. J.* **2010**, *99*, 1292–1302.

(14) Schäffer, E.; Nørrelykke, S. F.; Howard, J. Surface forces and drag coefficients of microspheres near a plane surface measured with optical tweezers. *Langmuir* **2007**, *23*, 3654–3665.

(15) Normanno, D.; Capitanio, M.; Pavone, F. S. Spin absorption, windmill, and magneto-optic effects in optical angular momentum transfer. *Phys. Rev. A* **2004**, *70*, 053829.

(16) van Oene, M. M.; Dickinson, L. E.; Pedaci, F.; Köber, M.; Dulin, D.; Lipfert, J.; Dekker, N. H. Biological magnetometry: Torque on superparamagnetic beads in magnetic fields. *Phys. Rev. Lett.* **2015**, *114*, 218301.

(17) Fonnum, G.; Johansson, C.; Molteberg, A.; Morup, S.; Aksnes, E. Characterisation of Dynabeads (R) by magnetization measurements and Mossbauer spectroscopy. *J. Magn. Magn. Mater.* **2005**, *293*, 41–47.

(18) Lipfert, J.; Hao, X.; Dekker, N. H. Quantitative modeling and optimization of magnetic tweezers. *Biophys. J.* **2009**, *96*, 5040–5049.

(19) Lipfert, J.; Wiggin, M.; Kerssemakers, J. W. J.; Pedaci, F.; Dekker, N. H. Freely orbiting magnetic tweezers to directly monitor changes in the twist of nucleic acids. *Nat. Commun.* **2011**, *2*, 439.

(20) Caizer, C.; Savii, C.; Popovici, M. Magnetic behaviour of iron oxide nanoparticles dispersed in a silica matrix. *Mat. Sci. Eng. B* **2003**, *97*, 129–134.

(21) Phillips, R.; Kondev, J.; Theriot, J.; Garcia, H. G.; Orme, N. *Physical Biology of the Cell*; Garland Science, 2012.
